# Triboelectric Nanogenerators Promote Self-Powered Sensing and Intelligent Monitoring

**DOI:** 10.3390/s26102984

**Published:** 2026-05-09

**Authors:** Yingxuan Cui, Tao Yang, Hongchun Luo, Yusheng Zheng

**Affiliations:** 1 School of Mechanics and Transportation Engineering, Northwestern Polytechnical University, Xi’an 710072, China; 2 Faculty of Science, Yibin University, Yibin 644007, China

**Keywords:** triboelectric nanogenerators, energy harvesting, self-powered sensing, intelligent monitoring

## Abstract

Against the backdrop of global energy structure decarbonization, distributed transformation, and the rapid development of low-power electronic devices and sensor networks, micro-energy supply and intelligent sensing have emerged as critical bottlenecks limiting their large-scale application. Triboelectric nanogenerators (TENGs), leveraging advantages such as compatibility with diverse materials and adaptability to flexible and miniaturized fabrication, can efficiently harvest widely available low-frequency, low-amplitude distributed mechanical energy in the environment. Additionally, they exhibit self-powered sensing characteristics, where output signals are directly correlated with external physical quantities, demonstrating unique strengths in the fields of micro-/nano-energy and intelligent monitoring. This article systematically reviews the research progress in TENGs; elucidates their working modes and power generation principles; summarizes material design, structural optimization, and performance enhancement strategies for efficient energy harvesting; and outlines the current state of self-powered sensing technologies. It highlights their engineering applications in intelligent monitoring scenarios such as drones, marine environments, infrastructure, and wearable devices. Addressing the existing technical bottlenecks and theoretical challenges in integrated energy harvesting–sensing–monitoring systems, the paper envisions future trends toward high performance, integration, and intelligence, providing valuable insights for fundamental research on and engineering applications of TENGs in micro-energy supply and intelligent monitoring.

## 1. Introduction

Against the backdrop of the global energy structure transitioning toward cleaner and more distributed systems, along with the deep integration of the Internet of Things (IoT), artificial intelligence (AI), and micro-nanoelectronics, micro-energy supply and intelligent sensing have emerged as critical challenges limiting the large-scale application of low-power electronic devices and distributed sensor networks [1,2,3]. On one hand, the depletion of traditional fossil fuels and increasingly stringent carbon emission regulations necessitate the urgent development of new clean and renewable energy technologies [4]. Low-frequency, low-amplitude distributed mechanical energy—such as environmental vibrations [5], human motion [6], water flow fluctuations [7], mechanical vibrations [8], and micro-wind tremors [9]—is widely available in nature, abundant in reserves, and easily accessible, making it a highly promising source of micro-energy. Its efficient harvesting and conversion have thus become a key research focus in energy engineering [10].

On the other hand, fields like intelligent manufacturing [11], intelligent cities [12], environmental monitoring [13], and structural health monitoring for engineering projects have led to a surge in demand for vast numbers of low-power sensors [14,15,16]. These sensors are often deployed in dispersed, mobile, enclosed, or hard-to-wire environments, where traditional wired power supply and battery-based solutions face issues such as high maintenance costs, limited endurance, and environmental pollution [17,18]. The development of self-powered sensing and intelligent monitoring technologies has thus become pivotal in overcoming these bottlenecks [19,20].

Depending on the different conversion mechanisms of mechanical energy, mechanical energy harvesting systems encompass various types, among which piezoelectric, electromagnetic, and triboelectric are three representative energy conversion methods. Piezoelectric vibration energy harvesting operates based on the piezoelectric effect, where environmental vibrations apply pressure to piezoelectric materials, causing strain in the piezoelectric layer. This induces the separation of positive and negative charges within the material, influencing the movement of surface charges to form an electric current for power output [21]. This method offers advantages such as a high output voltage, simple fabrication processes, ease of miniaturization and integration, and flexibility. It has been widely applied in portable devices, implantable devices, microelectromechanical systems (MEMS), and other fields. However, it also has inherent drawbacks, including high capacitive impedance, an extremely low output current, susceptibility to fatigue, and a service life limited by the properties of piezoelectric materials [22]. Electromagnetic vibration energy harvesting functions based on Faraday’s law of electromagnetic induction. Its core principle involves the relative motion between a conductor and a magnetic field to cut magnetic flux lines, generating an induced electromotive force that outputs electrical energy [23]. This conversion mechanism boasts advantages such as simple fabrication, a stable structure, and a relatively high output current and power, making it suitable for low-frequency working environments. However, traditional electromagnetic vibration energy harvesting systems are constrained by spatial limitations, making it difficult to achieve high output power at small scales, and they face challenges in miniaturization and integration [24].

The emergence and development of triboelectric nanogenerators (TENGs) have provided a solution to the aforementioned challenges [25]. Based on the coupling effect of triboelectrification and electrostatic induction, this technology has rapidly become a research hotspot across materials science, mechanical engineering, electronic engineering, energy engineering, and intelligent monitoring, owing to its core advantages such as high energy conversion efficiency at low frequencies, a simple structure, low fabrication costs, a broad material selection range, and integrated sensing–power generation capabilities [26,27].

Unlike traditional energy harvesting technologies, TENGs can flexibly utilize various substrates, including polymers, metals, biological materials, and inorganic materials, supporting flexible, transparent, stretchable, miniaturized, and array-based fabrication [28]. They are well suited for collecting low-frequency mechanical energy from diverse scenarios, such as environmental kinetic energy [29], micro-vibrations in engineering structures [30], human motion [31], and ocean microcurrents [32]. More importantly, the power generation process of TENGs is directly linked to changes in external physical quantities—their output electrical signals can directly reflect parameters like pressure [33], sound [34], displacement [22], and gas concentration [35], inherently enabling self-powered sensing without the need for additional power supply. This makes them perfectly aligned with the demands of intelligent monitoring [36].

After over a decade of development, TENGs have evolved from fundamental research to integrated applications in energy harvesting [37], self-powered sensing [38], and intelligent monitoring [39], emerging as a key technology for achieving self-sustaining micro-energy and intelligent perception [40,41].

Currently, TENGs have achieved numerous groundbreaking advancements in the fields of energy harvesting, self-powered sensing, and intelligent monitoring. In terms of energy harvesting, through surface material modification [42,43], biomimetic structural design [44], nonlinear dynamic regulation [45,46], and array integration [47], TENGs have enabled significant improvements in output power, energy conversion efficiency, and broadband adaptability [48,49]. They now enable the efficient collection of various low-frequency mechanical energies, such as wind vibrations [50], micro-vibrations in engineering structures, blue energy from the ocean [7], and human motion, providing stable micro-energy supplies for low-power sensors and portable electronic devices [51].

In the realm of self-powered sensing, TENG-based sensors for pressure, vibration, displacement, acceleration, and flow velocity combine high sensitivity [52], a wide detection range [53], and low detection limits with simple structures and low costs [54], eliminating the reliance on traditional power sources [19]. In intelligent monitoring, TENGs have been successfully applied in scenarios such as structural health monitoring, environmental monitoring, intelligent manufacturing [55], and human physiological signal monitoring [56], shifting from “passive powered monitoring” to “active self-powered monitoring” [16]. This offers a novel approach to building distributed sensor networks and intelligent monitoring systems [57]. Furthermore, the continuous improvement in integrating TENGs with energy storage devices, signal processing modules, and wireless transmission modules has further promoted their practical application in real-world engineering projects [58].

Based on this, this paper presents a systematic review of TENGs, highlighting their integrated development and application breakthroughs in three major fields: energy harvesting, self-powered sensing, and intelligent monitoring. Section 2 introduces the types and power generation principles of TENGs. Section 3 summarizes recent material designs, structural optimizations, and performance enhancement strategies for TENGs in efficient energy harvesting. Section 4 details the technological advancements in their application to self-powered sensor development. Section 5 provides an in-depth analysis of their practical applications in intelligent monitoring areas such as drone-based monitoring, marine environment monitoring, infrastructure monitoring, and wearable device monitoring. In the Discussion and Conclusions, the paper examines the core technical bottlenecks and theoretical challenges currently faced by TENGs in the integrated application of energy harvesting, sensing, and monitoring. Finally, considering the trends in disciplinary development and engineering application needs, it outlines the future prospects of TENGs toward high performance, integration, and intelligence, aiming to provide a reference and insights for fundamental research and engineering applications in this field and to promote TENGs as a core supporting technology in micro-energy supply and intelligent monitoring.

## 2. Power Generation Principles of TENGs

**Figure 1:**
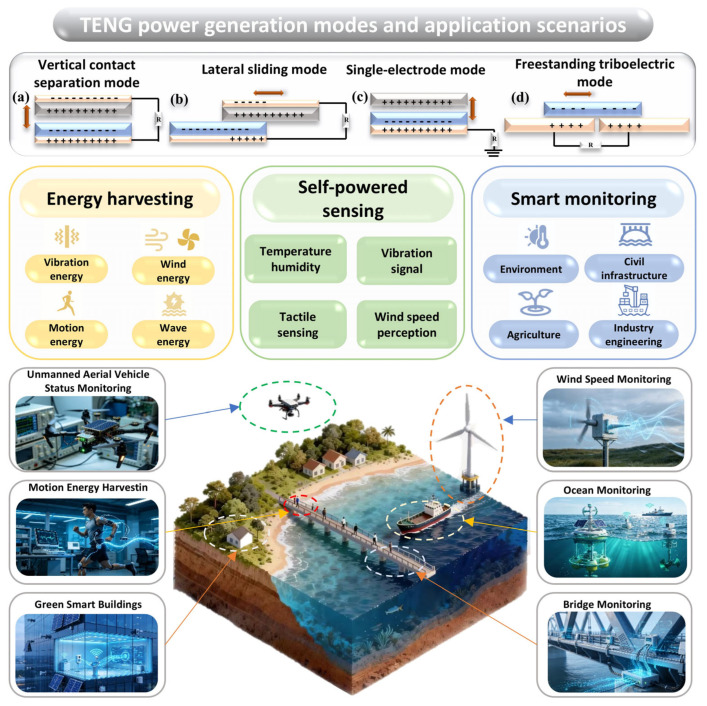
Categories, working principles, and basic application scenarios of TENGs.

As a novel energy harvesting technology, TENGs primarily encompass four core power generation modes, namely vertical contact separation mode, lateral sliding mode, single-electrode mode, and freestanding triboelectric layer mode, as shown in Figure 1. These modes efficiently capture widely distributed low-frequency and low-amplitude mechanical energy in nature through different interfacial motions and charge induction mechanisms.

Building on this technological foundation, TENGs can achieve the efficient harvesting and conversion of various clean energy sources in the field of energy harvesting, including vibration energy, wind energy, motion energy, and wave energy. In the realm of self-powered sensing, TENGs leverage the direct correlations between their output electrical signals and external physical quantities to enable the passive sensing of multiple parameters, such as temperature, humidity, vibration signals, tactile feedback, and wind speeds. In the domain of intelligent monitoring, TENGs find application in environmental monitoring, civil infrastructure monitoring, agricultural monitoring, and industrial engineering monitoring. Specific implementations include drone status monitoring, human motion energy harvesting and gait analysis, green intelligent building monitoring, wind speed monitoring, marine environment monitoring, and bridge structural health monitoring. These applications highlight the broad prospects and core value of TENGs in micro-energy supply, self-driven sensing, and intelligent monitoring.

As an emerging energy harvesting technology, TENGs can convert weak mechanical stimuli from the environment into electrical signals with voltage levels reaching volts. A rigorous mathematical formulation has been established based on Maxwell’s displacement current theory [59,60]. Its basic mathematical form can be expressed as

(1) $$\left\{\begin{array}{l} 𝛻\cdot D=\rho \\ 𝛻\cdot B=0\\ 𝛻\times E=-\frac{\partial B}{\partial t}\\ 𝛻\times H=J+\frac{\partial D}{\partial t} \end{array}\right.$$

where 𝛻 represents the vector differential operator; D represents the electric displacement vector; B and E, respectively, represent the magnetic field and the electric field; H is the magnetizing field; and $\frac{\partial D}{\partial t}$ represents the displacement current. This was introduced by Maxwell in 1861 to demonstrate the continuity of electric currents. The displacement current can also be expressed as

(2) $$D=\varepsilon_{0}E+P$$

where P represents the polarization field density, and $\varepsilon_{0}$ represents the vacuum permittivity. In a uniform, linear, and isotropic dielectric medium, the vacuum permittivity $\varepsilon_{0}$ can be expressed as the dielectric constant of the medium ε. Therefore, the displacement current $J_{D}$ can be further expressed as

(3) $$J_{D}=\frac{\partial D}{\partial t}=\varepsilon \frac{\partial E}{\partial t}+\frac{\partial P}{\partial t}$$

Wang introduced an additional charge polarization density $P_{s}$ to extend the traditional definition of the electric displacement vector ***D***. For the first time, a theoretical framework capable of comprehensively describing electromagnetic phenomena in moving media was established, redefining the electric displacement vector ***D*** as

(4) $$I_{D}=\frac{\partial D}{\partial t}=\varepsilon_{0}E+P+P_{s}$$

where the first term, the polarization vector ***P***, represents the linear electric polarization induced by the external electric field. The newly introduced charge polarization density term $P_{s}$ is independent of the electric field and results from the coupling of two physical mechanisms: triboelectricity and electrostatic induction.

After this, Maxwell’s equations can be written as

(5) $$\left\{\begin{array}{l} 𝛻\cdot I_{D}=\rho^{\prime }\\ 𝛻\cdot B=0\\ 𝛻\times E=-\frac{\partial B}{\partial t}\\ 𝛻\times H=J^{\prime }+\frac{\partial {I^{\prime }}_{D}}{\partial t} \end{array}\right.$$

where $I_{D}=\varepsilon_{0}E+P$. At the same time, by redefining the volume charge density $\rho^{\prime }$ and current density $J^{\prime }$, we obtain

(6) $$\left\{\begin{array}{l} \rho^{\prime }=\rho -𝛻\cdot P_{s}\\ J^{\prime }=J+\frac{\partial P_{s}}{\partial t} \end{array}\right.$$

Based on the principle of current conservation and extension equations $𝛻\cdot J^{\prime }+\frac{\partial \rho^{\prime }}{\partial t}=0$, the extended Maxwell’s displacement current $J_{D}$ can be expressed as

(7) $$J_{D}=\frac{\partial D}{\partial t}+\frac{\partial P_{s}}{\partial t}=\varepsilon \frac{\partial E}{\partial t}+\frac{\partial P_{s}}{\partial t}$$

In this extended theory, the displacement current density is defined as the sum of two terms. The first term $\varepsilon \frac{\partial E}{\partial t}$ corresponds to the conduction current generated by a varying electric field. It reveals the essence of how changes in electric fields can generate magnetic fields, serving as the physical foundation for deriving electromagnetic wave equations and playing a dominant role in high-frequency applications. The second term $\frac{\partial P_{s}}{\partial t}$ is the polarization current excited by the change in the distribution of static charges on the medium surface. It is directly related to mechanical motion and serves as the core physical mechanism for TENGs to convert mechanical energy into electrical energy under low-frequency conditions. 

The core working mechanism of the TENG lies in the fact that the displacement current within its internal medium connects and equals the conduction current in the external circuit at the electrodes, forming a complete loop for energy conversion. Based on effects such as piezoelectricity and triboelectricity, it utilizes the displacement current as the direct driving force to convert mechanical energy into electrical energy. This forms a conceptual contrast with traditional electromagnetic generators dominated by conduction currents, yet both are unified under the theoretical framework of Maxwell’s equations.

## 3. Energy Harvesting by TENGs

The long-term stable operation of wireless sensor networks is constrained by the lifespan and maintenance bottlenecks of traditional battery power, making environmental energy harvesting technology a key solution to overcome this limitation. Since the team led by academician Zhonglin Wang introduced the TENG in 2012 [61], TENGs, based on the principle of the displacement current, have demonstrated significant advantages in low-frequency and disordered mechanical energy harvesting, such as a high power density, broad material selection, and lightweight structure. These advantages address the shortcomings of traditional electromagnetic and piezoelectric power generation technologies in terms of low-frequency response, providing a highly promising technical pathway for powering micro-/nanoelectronic devices and constructing self-powered intelligent systems [62].

To further enhance the efficiency in capturing environmental vibration energy and broaden the effective operating frequency band, researchers have conducted extensive innovative explorations in the structural design of TENGs tailored to the vibration characteristics of different application scenarios [63]. Currently, mainstream vibration energy harvesting structural designs primarily include the following.

**Figure 2:**
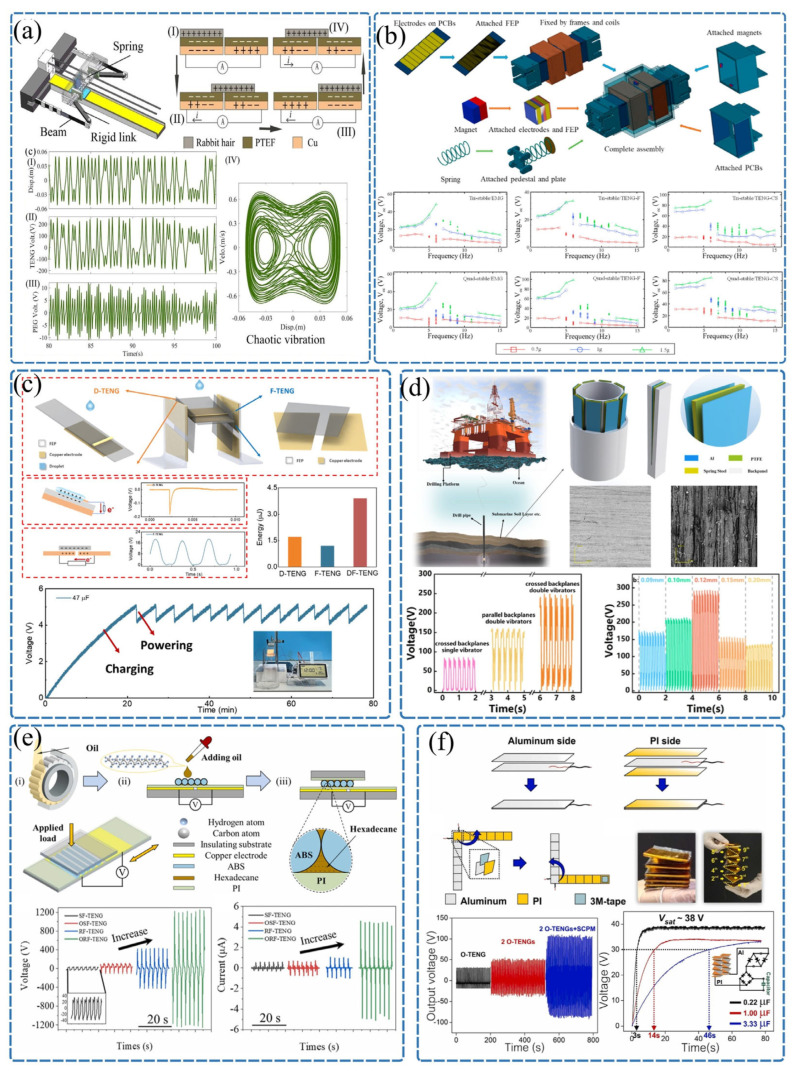
TENG-based energy harvester. (**a**) Bistable piezoelectric–triboelectric hybrid generator [64], (**b**) multi-stable vibration energy harvester [65], (**c**) flexible FEP cantilever beam TENG [66], (**d**) spring steel cantilever beam structure TENG [67], (**e**) oil-enhanced rolling TENG [68], (**f**) three-dimensional multi-layer origami structure TENG [69].

As illustrated in Figure 2a, Cui et al. [64] proposed a bio-inspired jellyfish-inspired low-barrier bistable piezoelectric–triboelectric hybrid generator, which utilizes springs, flexible beams, and rigid linkages to construct a geometrically nonlinear structure, enabling low-frequency broadband vibration energy harvesting at 3.5–6.5 Hz. The TENG module achieves a maximum output of 1050 V and 1.84 mW, capable of powering 70 LEDs. Both theoretical and experimental results validate its advantages of a low energy barrier, easy inter-well transition, and large amplitude, making it suitable for self-powering wireless sensors in bridge health monitoring systems.

As shown in Figure 2b, to tackle the challenge of energy harvesting in ultra-low-frequency environments, Yang et al. [65] proposed a spring-assisted multi-stable vibration energy harvester. This device innovatively combines electromagnetic and triboelectric generation mechanisms and effectively reduces the potential well depth by introducing a spring-based multi-stable nonlinear system. As a result, it achieves an impressive peak power density of 2261.3 W/m^3^ within the ultra-low-frequency excitation range of 1–5 Hz.

In the exploration of cantilever beam applications, as depicted in Figure 2c, Qu et al. [66] proposed a dual-function TENG (DF-TENG) based on a flexible fluorinated ethylene propylene (FEP) cantilever beam. By integrating droplet contact electrification with impact-induced beam vibration, the device synergistically captured both electrostatic energy and kinetic energy from droplets, increasing the charge output by approximately 1.9 times compared to single-mode operation. As illustrated in Figure 2d, addressing the power supply challenges in deep-well drilling, Lian et al. [67] developed a TENG based on a spring steel cantilever beam structure to harvest lateral vibration energy from drill pipes. This study successfully achieved omnidirectional energy harvesting within the two-dimensional plane inside the drill pipe, with the system’s peak power reaching 30.95 mW under typical working conditions, providing a novel solution for powering downhole intelligent measurement systems.

As shown in Figure 2e, to address the limitations imposed by friction and wear on the device lifespan, Wang et al. [68] were inspired by cylindrical roller bearings and developed an oil-enhanced rolling friction TENG (ORF-TENG). This design leverages the synergistic effect of rolling friction and oil molecules, reducing the friction coefficient by 84.9% while increasing the open-circuit voltage and energy conversion efficiency by 32.1 times and 263 times, respectively. As illustrated in Figure 2f, to achieve cost-effective and scalable high-efficiency energy harvesting, Pongampai et al. [69] proposed a three-dimensional multi-layer origami-structured TENG (O-TENG) integrated with a self-charging pumping module. Through the co-optimization of the origami structure and surface roughness modification, this design significantly enhanced the output performance, achieving a maximum power output of 697 μW under a 10 MΩ load, demonstrating its great potential as a self-powered source for portable electronic devices. Research on TENGs for vibration energy harvesting has achieved significant progress in the integration of structural topology optimization and working mechanisms [70]. By introducing innovative solutions such as spring–mass multi-stable mechanisms, flexible cantilever coupling structures, rolling friction, and multi-layer origami designs, researchers have successfully addressed the adaptability challenges of TENGs in broadband, multi-dimensional, and random low-frequency vibration environments. Meanwhile, the development of hybrid power generation modes has further broken through the power density bottlenecks of single mechanisms. These cutting-edge technologies have demonstrated broad application prospects in various fields, including marine pipeline monitoring, deep-well drilling, rail transit safety, and power supply for portable electronic devices, fully validating the feasibility of TENGs as a distributed micro–nano energy solution.

In the pursuit of high-performance TENGs, circuit design and system-level integration have emerged as pivotal factors complementing material and structural innovations. As demonstrated in recent studies, advanced circuit architectures—ranging from multi-electrode switching and self-charge pumping to external capacitive regulation and precision signal conditioning—play a critical role in optimizing output characteristics, ensuring operational safety, and expanding application scenarios. These works highlight how tailored circuit solutions can address key challenges such as charge accumulation, voltage breakdown, and measurement inaccuracy, thereby unlocking new possibilities for TENGs in both energy harvesting and intelligent monitoring.

**Figure 3:**
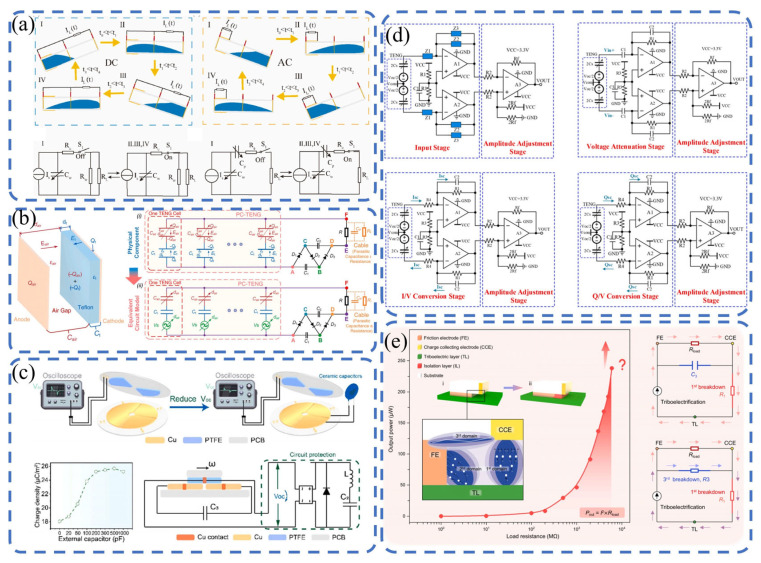
Strategies for enhancing TENG performance. (**a**) Four-electrode tubular liquid–solid TENG [71], (**b**) self-charge pumping circuit with high-reverse-voltage diodes [72], (**c**) TENG with enhanced charge density and limited open-circuit voltage [73], (**d**) configurable high-precision multi-parameter signal measurement method and circuit framework [74], (**e**) impedance matching rules and voltage–current/charge regulation strategies [75].

As shown in Figure 3a, Zhang et al. [71] proposed a four-electrode dual-mode tubular liquid–solid TENG (DMTLS-TENG) with switchable AC/DC outputs. At an FEP tube length of 15 cm, the transferred charge per cycle reached 200 nC, one order of magnitude higher than that in AC mode. The regulation mechanisms of the water volume ratio, tube length, and ion concentration on DC/AC outputs were revealed. The DMTLS-TENG shows potential applications in oil–water separation and corrosion resistance, providing a new route for large-scale applications of LS-TENGs. Currently, the development of high-performance TENGs faces bottlenecks due to the lack of quantitative analysis methods for their solid–liquid energy conversion processes. Zheng et al. [29] proposed a quantitative analysis method called Kinetic Energy Calculation and Current Integration (KECCI), which significantly enhances the understanding of the mechanical-to-electrical energy conversion process. Through the systematic optimization of bionic surface structures and instantaneous switch designs, the energy conversion efficiency of TENGs reached up to 24.89%. Additionally, the multi-layer structural design enables the continuous harvesting of raindrop kinetic energy.

As illustrated in Figure 3b, Yin et al. [72] investigated the effect of a self-charge pumping circuit (SCPC) on stacked TENGs and found that high-reverse-voltage diodes can induce excessive charge accumulation and corona discharge. A stepwise charge enhancement strategy was proposed, increasing the short-circuit transferred charge from 50.2 nC to 69.4 nC and finally to 94.0 nC. The method is safe, simple, and allows the flexible tuning of the triboelectric charge polarity, with edge breakdown systematically studied, supporting the design of high-performance TENGs. As presented in Figure 3c, Zhang et al. [73] introduced a strategy using an external capacitor C3 to simultaneously enhance the charge density and limit the open-circuit voltage (Voc < 1000 V), avoiding breakdown in the power management circuit (PMC). With C3 = 100 pF, the PMC efficiency was improved by 1.7 times. After optimizing the inductance and capacitance, record-high efficiency of 51.8% was achieved at 5 V. The self-powered system was successfully applied in industrial environmental monitoring. As shown in Figure 3d, Jiang et al. [74] developed a configurable high-precision signal characterization method and a fully differential amplification circuit framework for TENGs, enabling accurate voltage, current, and charge measurements. The circuit adopted a capacitively coupled instrumentation amplifier with high input impedance and strong anti-interference abilities. The voltage measurement error was reduced from 48.2% to 3%, and the current and charge errors were controlled within 5% and 6%, respectively, supporting standardized TENG characterization. As illustrated in Figure 3e, Zhang et al. [75] established a load-oriented operating framework for a direct-current TENG (DC-TENG), revealing how load resistance regulates electrostatic breakdown and the equivalent internal resistance. Impedance matching rules and voltage–current/charge regulation strategies were proposed and verified. The constructed DC-TENG delivered a maximum Voc of 25 kV and nearly constant current from short circuit to 74 GΩ, supporting the development of high-precision high-impedance power systems.

The future of TENG performance enhancement lies in the seamless integration of innovative materials, optimized structures, and sophisticated circuit designs. From the four-electrode dual-mode configuration that enables switchable AC/DC outputs to self-charge pumping strategies that safely boost the transferred charge, and from external capacitor-assisted power management achieving record efficiency to high-precision differential amplification for accurate characterization, each approach contributes to a more robust and versatile TENG technology platform. Notably, during the process of converting mechanical energy into electrical energy, the amplitude–frequency characteristics of the TENG’s output electrical signals are directly controlled by the motion state of the external excitation source. This inherent electromechanical response correlation enables the TENG to accurately map the physical attributes of vibration sources without requiring an external power supply, thereby laying the physical foundation for its evolution from a simple energy harvester to a self-powered sensor [76].

The enhancement of energy harvesting capabilities directly overcomes the inherent limitations of traditional TENG sensors in terms of the sampling rate, signal-to-noise ratio, and duty cycle by providing a more stable and continuous power supply. This not only improves the real-time performance and detection accuracy in sensing signals but also provides energy headroom for integrating functional modules such as signal processing and wireless transmission modules, fundamentally optimizing the performance boundaries and engineering application potential of TENG sensors.

## 4. Self-Powered Sensing Based on TENGs

**Figure 4:**
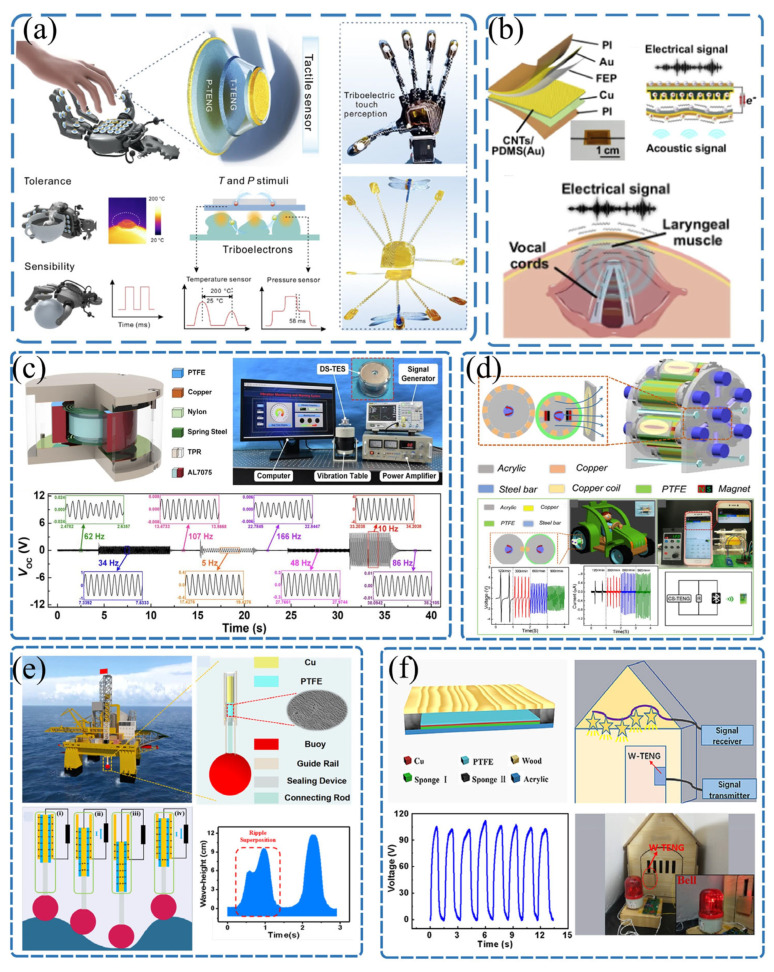
Different types of self-powered TENG sensors. (**a**) Triboelectric tactile sensor for pressure and temperature sensing [33], (**b**) triboelectric acoustic sensor [34], (**c**) vibration and acceleration sensing TENG [16], (**d**) rotation speed and displacement sensing TENG [84], (**e**) ocean wave parameter and wave velocity spectrum sensing TENG [85], (**f**) human motion trajectory sensing TENG [86].

**Figure 5:**
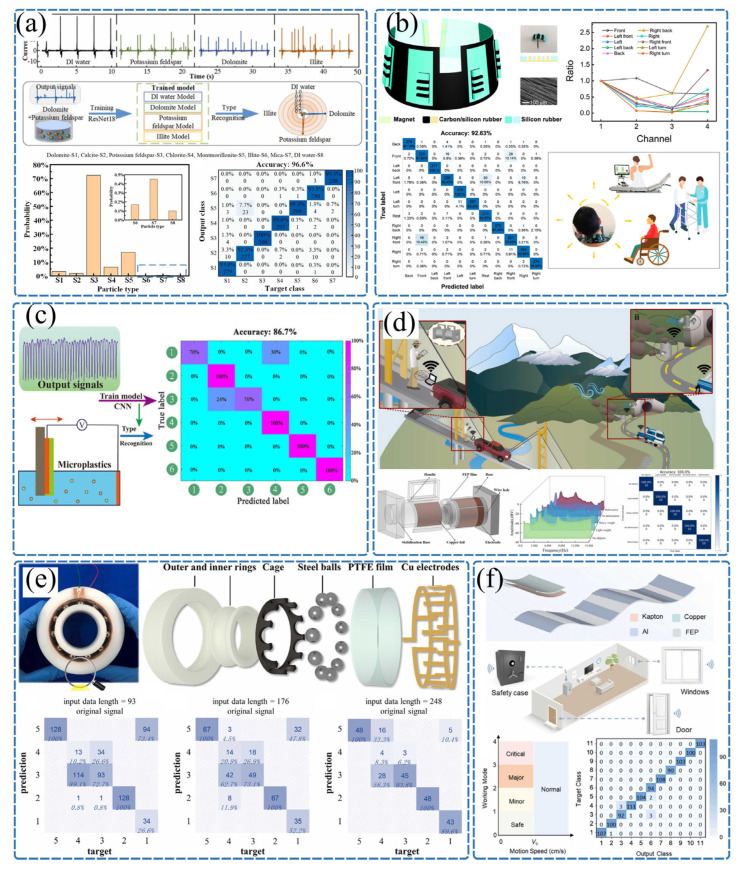
TENG sensing applications combined with machine learning. (**a**) TENG for sediment particle identification [39], (**b**) TENG for neck motion posture recognition [87], (**c**) TENG for microplastic classification and identification [88], (**d**) TENG for bridge component deformation and risk monitoring [89], (**e**) TENG for bearing sphere defect identification [90], (**f**) TENG for whole-house intelligent security [91].

Traditional sensing technologies generally rely on external power sources to modulate and measure physical quantities. This passive operational mode significantly limits their application in extreme environments or large-scale distributed networks [77,78]. In contrast, a TENG is not only an efficient micro/nano energy device but also a natural active sensor. Based on Maxwell’s displacement current principle, there is an intrinsic functional relationship between the electrical output signals of the TENG (such as the open-circuit voltage, short-circuit current, and transferred charge) and the external mechanical excitation parameters (such as displacement, velocity, acceleration, and contact pressure) that induce charge transfer [79,80]. This means that, without any external driving circuit, a TENG can directly and in real time map mechanical physical quantities from the environment into detectable electrical signals, offering significant advantages such as zero static power consumption, a high signal-to-noise ratio, a wide dynamic response range, and millisecond-level transient capture capabilities [81,82,83]. For example, the working principle of the contact-separation triboelectric nanogenerator is illustrated in Figure S1, and the relationship between voltage, current, and displacement, along with the derivation process, can be found in the Supplementary Materials. Thanks to these advantages, TENGs have transcended the scope of mere energy harvesting and have been widely applied to the in situ monitoring of various complex physical fields, as shown in Figure 4. With the rapid development of artificial intelligence technology, the deep integration of TENGs’ rich waveform information with machine learning algorithms has become a key approach to endowing sensors with “intelligence”, as illustrated in Figure 5.

As shown in Figure 4a, Liu et al. [33] developed a multimodal triboelectric sensor capable of adapting to extreme environments, which can detect pressure and temperature signals beyond the range of human perception. Based on triboelectric nanogenerator technology, the research designed an asymmetric structure capable of independently outputting dual signals, effectively enhancing the sensing sensitivity. By converting signals and stimuli into feature matrices, the sensor achieved the parallel perception of complex objects (with a recognition rate of 94%) and temperature detection in high-temperature environments. This multimodal triboelectric tactile sensor made breakthroughs in the maximum detection range and rapid response, surpassing the upper limit of human skin’s high-temperature perception (60 °C) and operating at temperatures of up to 200 °C. As presented in Figure 4b, Yao et al. [34] developed an anti-noise triboelectric acoustic sensor (anti-noise TEAS) based on a flexible nanopillar structure and integrated it with a deep learning model based on convolutional neural networks (anti-noise TEAS-DLM), constructing a highly synergistic sensing system that enables stable acoustic signal recognition in complex and noisy environments, ensuring smooth human–machine collaboration. The anti-noise TEAS directly captures the fundamental frequency signals of mixed-mode vibrations in the throat through contact sensing, while effectively suppressing environmental noise interference by optimizing the device’s structural buffering design. Subsequently, the deep learning model processes and semantically decodes the acoustic signals, ensuring high-fidelity interpretation. Verified through simulated virtual and real noisy environment tests, the anti-noise TEAS-DLM system exhibits near-perfect noise resistance, reliably transmitting various voice commands and accurately guiding robotic systems to perform complex post-disaster rescue tasks.

Zhang et al. [35] reported an eco-friendly self-powered nitrogen dioxide (NO_2_) sensor (EFNS) system based on an environmentally friendly triboelectric nanogenerator (EF-TENG). The system consists of a power supply unit (EF-TENG) and a sensing unit (In_2_O_3_/PPy sensor), connected via a signal stabilization circuit. The EF-TENG uses environmentally friendly and biodegradable gelatin and PLA/PBAT as triboelectric layers, increasing the output voltage and current by 1.44 and 1.67 times, respectively, with the peak and root mean square power densities reaching 1386 mW·m^−2^ and 185.35 mW·m^−2^. Under conditions of 15–55 °C and 40–80% relative humidity, it maintains a stable output voltage (approximately 24 V) after rectification and regulation. Meanwhile, the study fabricated an In_2_O_3_/PPy heterostructure sensor and constructed the EFNS system through impedance matching effects, elucidating the response mechanism of the In_2_O_3_/PPy heterostructure to NO_2_ and achieving the wide-range, high-sensitivity detection of NO_2_ (Vg/Va = 355% at 30 ppm).

As illustrated in Figure 4c, in the field of high-precision vibration and acceleration measurement, Wang et al. [16] proposed a double-spring triboelectric sensor (DS-TES) for broadband vibration monitoring. This device utilizes a dual three-rib longitudinal spring structure to establish a stable internal spring–mass–damping system, achieving a wide frequency response range of 0–200 Hz while maintaining exceptional measurement linearity, with a frequency error rate below 0.015%. It demonstrates promising potential for applications in natural disaster early warning systems and real-time structural health monitoring. As illustrated in Figure 4d, to address the monitoring needs of rotating equipment in extreme environments, Yang et al. [84] developed a hybrid nanogenerator based on rolling friction and contact separation modes. This device exhibits remarkable durability, maintaining 96% output retention after continuous operation for two days. It successfully enables the self-powered real-time sensing of the rotational speed and displacement, providing a reliable passive condition monitoring solution for exploration equipment operating in harsh environments, such as lunar rovers.

As depicted in Figure 4e, to meet the demand for the precise acquisition of marine hydrological information, Zhang et al. [85] developed a self-powered wave spectrum sensor composed of a tubular TENG and a hollow spherical buoy. This device boasts ultra-high wave height detection sensitivity of 2530 mV mm^−1^ and an extremely low periodic monitoring error of 0.1%. It can accurately invert six key wave parameters (e.g., wave height, wave speed, wavelength) and wave speed spectra based on electrical signals, providing high-quality real-time data sources for marine big data analysis. As illustrated in Figure 4f, in the exploration of eco-friendly sensing materials, Hao et al. [86] developed a TENG based on natural biodegradable wood, capable of achieving a power density output of 158.2 mW/m^2^. It was applied in intelligent home scenarios as a self-powered switch and doorbell. Furthermore, by integrating the W-TENG into flooring, the study successfully constructed an intelligent stage capable of the real-time tracking of dancers’ movement trajectories and interactive lighting control, demonstrating the immense potential of TENGs in human activity monitoring and big data analytics.

The electrical signals generated by TENGs often contain extremely rich time–frequency-domain features, making it difficult for simple threshold detection to uncover their deeper meanings. By introducing feature engineering and pattern recognition algorithms, high-dimensional state information can be extracted from complex nonstationary signals. This enables the self-powered sensing system not only to detect changes in physical quantities but also to recognize complex behavioral patterns and operational conditions, providing robust algorithmic support for advanced applications such as human posture recognition, gait analysis, and equipment fault diagnosis.

As shown in Figure 5a, in the field of intelligent hydrological sensing and monitoring, Yu et al. [39] proposed a real-time sediment monitoring method that combines a particle-loaded droplet-driven TENG with a deep learning algorithm. By leveraging the high sensitivity of TENG output signals to suspended particle types and mass fractions, the method employs a convolutional neural network model to deeply analyze complex sensing electrical signal characteristics, achieving the high-precision identification and visual monitoring of sediment particle parameters in water bodies. As depicted in Figure 5b, in the field of human health monitoring and rehabilitation medicine, An et al. [87] developed a wearable neck motion monitor based on a flexible triboelectric sensor array and deep learning algorithms. By incorporating a carbon-doped silicone rubber shielding layer and a convolutional neural network model, the system successfully achieved the accurate recognition of 11 neck motion postures, including eight bending directions, two twisting directions, and a stationary state, with an average accuracy rate as high as 92.63%.

As shown in Figure 5c, to address the challenge of detecting environmental microplastic pollution, Huang et al. [88] proposed a novel detection method based on a liquid–solid TENG combined with deep learning. Experimental results confirmed that, within the mass fraction range of 0.025 to 0.25 wt%, the output voltage exhibited a highly linear correlation with the microplastic content. By integrating a convolutional neural network model, the high-precision classification and identification of five common microplastics, including polyethylene and polypropylene, were successfully achieved. As illustrated in Figure 5d, to meet the demand for multi-parameter monitoring in complex natural environments, Liu et al. [89] developed a deep learning-assisted self-powered wireless environmental monitoring system based on a rotary switch TENG. By incorporating a corona discharge structure and a resonant circuit, the system successfully enabled the wireless transmission of environmental data such as wind speed, bridge deformation, and mountain rockfall. Through the integration of deep learning algorithms, the host computer’s visualization interface can intelligently identify the deformation statuses of bridge components and assess rockfall risk levels in real time.

As shown in Figure 5e, to address the micro-fault diagnosis needs of rolling bearings in high-end equipment, Dong et al. [90] designed an embedded flexible thin-film triboelectric sensor for intelligent bearings and proposed a novel analytical framework combining signal decomposition and automated machine learning (AutoML) to process similar triboelectric signals generated by rolling element defects. The system significantly improved the identification accuracy for five different ball defect conditions from 78.34% to 99.48%. As illustrated in Figure 5f, for home security scenarios, Xu et al. [91] developed a ternary electrification layered triboelectric film sensor with excellent flexibility and transparency. By integrating deep learning algorithms, it achieved a classification recognition rate of up to 99.2% for various intrusion or operational actions. They also developed a companion mobile app, establishing a low-cost, visual, self-powered whole-house intelligent security monitoring system.

The deep integration of deep learning algorithms with TENG technology has successfully elevated self-powered sensing from the detection of single physical quantities to an intelligent cognition level for complex environments and working conditions. Whether in hydrological parameter inversion, human motion posture reconstruction, industrial equipment fault diagnosis, or intelligent security applications, it has demonstrated exceptionally high recognition accuracy and application robustness [92].

Research on TENGs for self-powered sensing revolves around two core aspects: self-powering and intelligent sensing. On the one hand, it focuses on developing adaptable sensors for various application scenarios, breaking the traditional reliance on external power sources. On the other hand, it integrates machine learning technologies to construct intelligent sensing algorithms, achieving a leap from signal acquisition to information comprehension. These advancements lay the foundation for the long-term autonomous operation of sensing systems. Moreover, the energy harvesting characteristics of TENGs and the precise sensing capabilities of self-powered sensing provide both power support and a data foundation for state recognition in monitoring scenarios.

## 5. Intelligent Monitoring Application Based on TENGs

TENGs are driving intelligent monitoring toward self-powering, high integration, and full-scenario use. With their strengths in energy harvesting and self-powered sensing, they offer an integrated solution for energy, sensing, and analysis. This section studies four key scenarios. In drone attitude monitoring, TENGs transform flight vibrations and airflow into electricity to run sensors that track attitude angles and acceleration. For marine environment monitoring, they use wave and current energy to power underwater sensors that measure temperature, salinity, and pressure, solving problems related to short battery lives and challenging deployment. In infrastructure monitoring, TENGs gather structural vibration energy to supply strain and crack sensors for long-term safety checks. In human health monitoring, they harvest motion energy to drive flexible patches that monitor heart rate, electromyography, and other signals, resulting in unobtrusive wearables. By examining these cases, this section shows how TENGs enable a closed loop of self-sustaining energy, precise sensing, and intelligent analysis, moving monitoring from a proof of concept to large-scale use.

### 5.1. Condition Monitoring for Low-Altitude Aircraft

**Figure 6:**
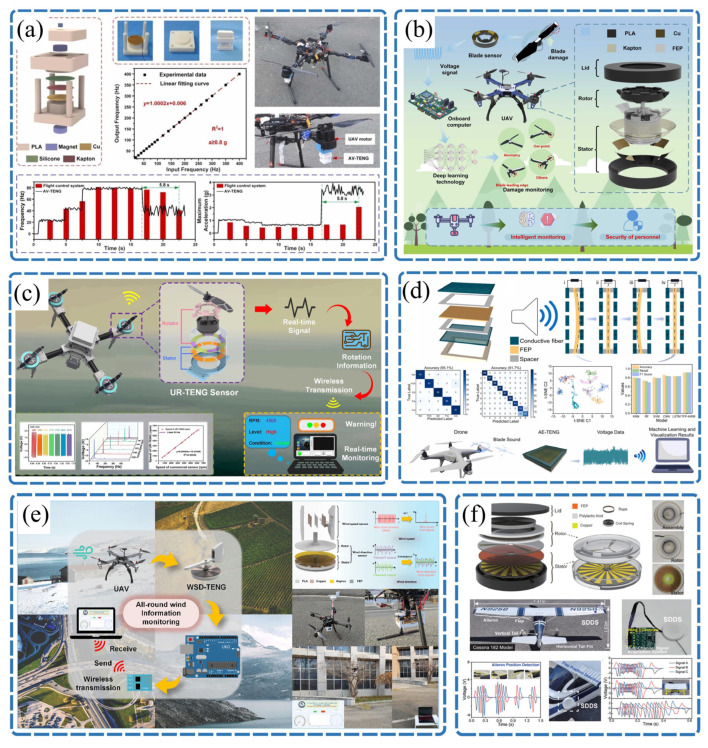
Related applications of TENGs in sensor detection for low-altitude aircraft. (**a**) Motor parameter monitoring and early warning TENG [94], (**b**) blade damage classification and identification TENG [95], (**c**) motor speed and abnormal state monitoring TENG [96], (**d**) fault classification and identification TENG [97], (**e**) wind speed sensing and monitoring TENG [98], (**f**) displacement sensing TENG [22].

In recent years, researchers have begun exploring the application of TENG technology in the aviation sector, aiming to convert harmful vibrational energy during flight into usable electricity while leveraging its self-powered sensing capabilities for the real-time monitoring of the rotor speed, aircraft attitude, and structural damage. This approach offers a highly promising technical solution for developing next-generation low-altitude flight platforms with extended endurance and intelligent features [93], as illustrated in Figure 6.

As illustrated in Figure 6a, addressing the lack of effective real-time monitoring methods for abnormal vibrations in drone motors, Wang et al. [94] proposed a self-powered vibration sensor (AV-TENG) based on a magnetic spring structure. By utilizing the non-contact nature of magnetic forces to replace traditional mechanical springs, it achieves exceptional linearity in frequency detection across a broad bandwidth (with a maximum error of only 0.0062%). This innovation successfully enabled the real-time wireless monitoring of the vibration frequency and acceleration in drone motors during flight tests, along with early fault warnings. Figure 6b demonstrates another breakthrough: to tackle the challenge of the real-time, precise identification of drone blade damage, Pan et al. [95] developed a blade damage monitoring system integrated with a deep learning algorithm. The system collects rotational signals via a triboelectric sensor installed on the motor and employs a deep learning model to overcome the limitations of conventional frequency-domain analysis, which struggles to establish universal criteria. This system achieved impressive classification accuracy of up to 94.4% for different types of blade damage.

As shown in Figure 6c, to overcome the challenges of motor speed monitoring installation and power supply in confined spaces, Guan et al. [96] developed a wireless online monitoring system (UR-TENG) based on a soft-contact structure and independent layer mode. Utilizing a low-friction design, it effectively minimized the impact on the motor load while achieving ultra-high goodness of fit of 0.99 and a low error rate of only 0.014 within a speed range of up to 6270 rpm. Integrated with a microcontroller and WiFi module, the system successfully enabled the real-time wireless data transmission of the drone motor speed and abnormal state detection. As illustrated in Figure 6d, for blade health monitoring, Lu et al. [97] proposed an innovative non-contact fault detection method based on acoustic signals. The study employed a flexible triboelectric sensor as a highly sensitive acoustic–electric transducer to capture blade rotation noise. Combined with convolutional neural networks to deeply analyze the time–frequency features of the signals, it achieved classification accuracy of up to 95.1% for four common faults: cracks, fractures, foreign object adhesion, and edge deformation.

As depicted in Figure 6e, to address the issues of a bulky size and high power consumption in traditional airborne anemometers, Wang et al. [98] designed a WSD-TENG with a hierarchical structure. By integrating thin-film and disk configurations, the device successfully achieved omnidirectional adaptive monitoring across a wide flow velocity range of 2.6–30.0 m/s with a wind direction resolution of 5°. Its output frequency exhibited a linear correlation with the wind speed (R^2^ = 0.9995), providing a lightweight environmental sensing solution for stable drone flight in complex airflow conditions. As illustrated in Figure 6f, targeting the safety risks posed by the lack of position sensing in drone flight actuators, Zhou et al. [22] developed a self-powered digital displacement sensor (SDDS) for flight actuation systems. By leveraging triboelectric charge separation signals, it enabled the real-time monitoring of actuator position states, offering a potential solution to the incompatibility of traditional aviation-grade displacement sensors—due to their excessive size and weight—with micro-drone systems.

Self-powered sensing technology has demonstrated broad application prospects in the field of low-altitude aircraft condition monitoring [99]. Leveraging the flexibility in material selection and lightweight structural design advantages of TENGs, existing research has successfully validated their capabilities to sense key parameters such as rotor speeds, structural vibrations, and attitude changes without increasing the flight load burden. This provides a highly competitive technical pathway to resolve the conflict between power consumption and sensing in micro-aircraft. However, to truly achieve the transition from laboratory prototypes to engineering applications, current research must still overcome several significant challenges [100,101].

The primary bottleneck lies in the fact that the vibration signals generated during the dynamic operation of an aircraft often exhibit a highly nonlinear and nonstationary nature, frequently accompanied by the aliasing of multi-source signals. A sole reliance on traditional signal processing methods or simple threshold judgments makes it difficult to achieve the accurate interpretation of flight states [102]. More critically, research on the deep integration of self-powered sensing with artificial intelligence technology is still in its infancy, particularly in constructing lightweight, high-precision machine learning models tailored to the specific scenario of low-altitude aircraft—a gap that remains unaddressed. The lack of effective feature extraction and intelligent pattern recognition algorithms prevents vast amounts of sensor data from being transformed into real-time fault diagnosis decisions and safety warning information. This has become the core shortcoming hindering the realization of intelligent, autonomous monitoring in self-powered sensing systems and represents a crucial research direction for future breakthroughs [103,104,105].

### 5.2. Intelligent Monitoring of Marine Environment

The ocean environment features high humidity, high salinity, and strong corrosiveness, which often limit conventional electronic monitoring devices due to difficulties in power supply and high maintenance costs. TENG technology, with its self-powering capabilities and resistance to environmental interference, can convert mechanical energy from wave impacts and tidal movements into electricity while simultaneously performing multi-parameter monitoring. This creates new opportunities for long-term, unattended observation in marine environments.

**Figure 7:**
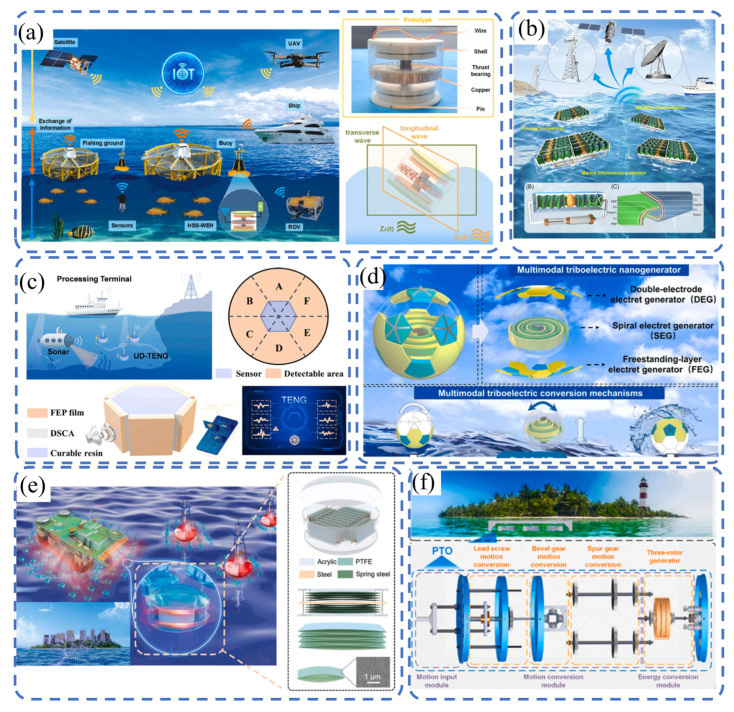
Applications of TENGs in marine environmental monitoring. (**a**) Hybrid self-powered and self-sensing wave energy harvester [106], (**b**) high-performance TENG based on double-spiral zigzag–origami structure for continuous sensing and signal transmission in marine environment [107], (**c**) application scenario and detailed structure diagram of UD-TENG [108], (**d**) AI-driven ocean monitoring with multimodal TENG [109], (**e**) TENG for efficient blue energy harvesting of all-sea areas [110], (**f**) self-sensing wave energy harvester based on three-rotor motor of axle disk type [38].

As shown in Figure 7a, Liu et al. [106] proposed a hybrid self-powered and self-sensing wave energy harvester (HSS-WEH) for powering sensors in sustainable fisheries and wave monitoring. It solves the problems of battery replacement and leakage pollution and is equipped with a self-monitoring module for real-time wave perception. In this study, an LSTM model was adopted for real-time ocean monitoring. The data were split into training and test sets using the sliding window method, and the wave hazard level was predicted via the SoftMax activation function. After 100 iterations, the training accuracy reached 98.62% with a loss rate of 3.56%. The confusion matrix showed that the recognition accuracy was 98% for safe sea areas and 97% for dangerous sea areas, demonstrating excellent accuracy and reliability. The HSS-WEH can simultaneously achieve wave monitoring and sensor power supply, supporting the development of IoT-based marine systems.

As illustrated in Figure 7b, Jiang et al. [107] designed a high-performance TENG (D-Z TENG) with a double-helix zigzag origami structure and developed a power management circuit integrating efficient energy harvesting and on-demand power supply. It can drive commercial sensors and construct self-powered IoT sensing nodes, realizing the integration of energy harvesting, data processing, and wireless transmission. This TENG can charge lithium batteries in natural waves and continuously power water quality sensors for signal transmission, laying a foundation for practical applications. As shown in Figure 7c, Guan et al. [108] proposed a compact and easily fabricated ultrasonic-driven TENG (UD-TENG) and provided its equivalent circuits in various scenarios. The system forms a self-charging power unit via a rectifier, charging a 1 mF capacitor to 1.5 V in approximately 20 s and directly driving 27 LEDs at 40 kHz. The peak power density reaches 4.5 mW/m^2^ at a 3 MΩ load. A thin-film sensor perpendicular to the acoustic wave direction and a grounded cavity effectively protect against leakage current and high-frequency electromagnetic interference.

As presented in Figure 7d, Mao et al. [109] proposed an AI-enhanced self-powered monitoring system (SAMS) based on a multimodal TENG for intelligent wave perception. The system adopts a soccer-ball-like packaging structure and integrates three power generation units: continuous liquid–solid contact (FEG), environmental vibration (SEG), and intermittent liquid–solid contact (DEG). Waves are classified into four intensity levels. Using a 2D-CNN for multimodal signal fusion recognition, the system’s accuracy is improved from 41.25% to 96.25%, providing a new strategy for wave monitoring in harsh environments. As illustrated in Figure 7e, Feng et al. [110] developed a half-meter-scale TENG (HM-TENG) for power sensing in coastal cities. The device adopts a multi-arch structure with a vertical central steel plate, optimizing space utilization and achieving full contact separation. Moreover, 7° inclination enables the stacked friction layers to operate over a wide frequency range of 0.1–2 Hz with excellent output performance. It charges a 47 μF capacitor to 5.5 V within 150 s and shows no performance degradation after 14 h of continuous operation, demonstrating outstanding robustness and guiding the large-scale development of blue energy. As shown in Figure 7f, Xia et al. [38] proposed a self-powered and self-sensing wave energy harvester (SS-WEH) with a three-rotor structure, which converts wave excitation into stable rotational motion. Under 1 Hz/50 mm excitation, it delivers average output power of 8.24 W at the optimal load of 600 Ω, with a power density of up to 675 W/m^3^, being superior to conventional electromagnetic harvesters. Combined with the GRU neural network for wave state classification, the training accuracy reached 99.56% after 50 iterations, enabling the high-precision recognition of five voltage states. This work integrates wave energy harvesting, environmental monitoring, and system self-diagnosis, providing a key technical route for self-powered sensing systems.

Integrating TENGs with sensors for temperature, salinity, depth, dissolved oxygen, and turbidity can produce marine monitoring nodes that combine energy harvesting and data acquisition, enabling multi-level deployment from coastal areas to deep seas. As waterproof encapsulation and corrosion-resistant materials continue to improve, TENG-based systems will become more robust in oceanographic intelligent monitoring, delivering sustained data support for resource development, ecological protection, and disaster early warning.

### 5.3. Intelligent Monitoring of Infrastructure

Bridges, tunnels, and high-rise buildings are subject to loads, temperature variations, and environmental erosion over their service lives, making timely health assessment critical. Conventional monitoring systems often require external power and involve complex wiring, limiting large-scale deployment. TENGs can generate electrical signals from mechanical energy such as structural vibrations and strain changes, thus enabling passive, distributed structural health monitoring.

**Figure 8:**
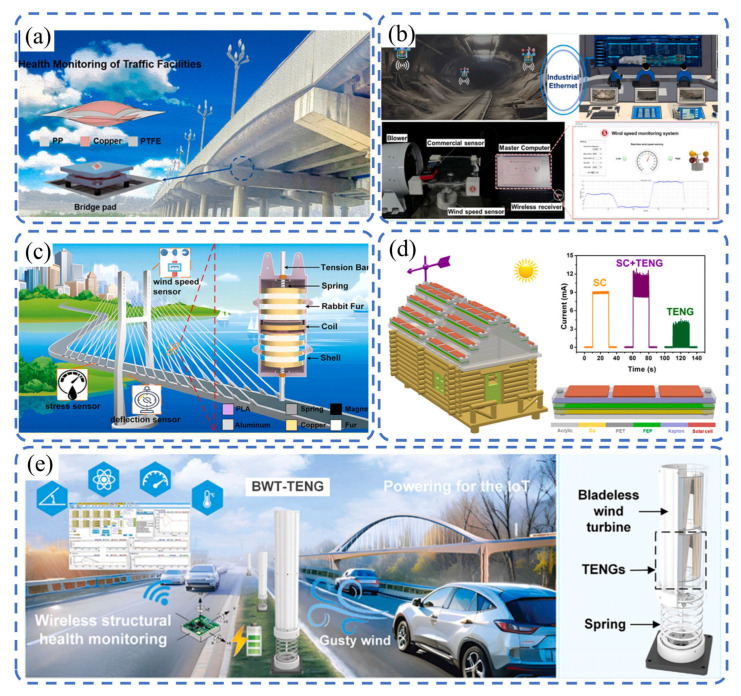
Applications of TENGs in infrastructure health monitoring. (**a**) The detailed structural design of the CS-TENG [111], (**b**) a self-powered wind speed sensing system based on the ASWS-sensor [53], (**c**) a cable vibrational energy harvesting network constructed by the CCT-TEHG [112], (**d**) the efficient scavenging of solar and wind energies in an intelligent city [113], (**e**) a bladeless wind turbine TENG for effectively harvesting random gust energy [114].

As depicted in Figure 8a, TENGs have been successfully applied to the condition monitoring of key bridge components such as cables and expansion joints. They can effectively capture structural vibrations induced by traffic loads or natural wind excitations, thereby realizing the real-time health assessment of bridge infrastructure. Xu et al. [111] proposed a capsule-shaped TENG (CS-TENG), which enables the self-powered detection of bridge deformation with high sensitivity of 1 mm. Without an external power supply, it can provide early warning for potential hazards such as bridge settlement, offering a novel monitoring method for the safe operation and maintenance of large-scale civil structures. As illustrated in Figure 8b, Yang et al. [53] combined a TENG with an electromagnetic generator (EMG) and designed a dual-cup structured composite sensor. This device can not only accurately measure the wind speed in the range of 1.5–15 m/s but also synchronously outputs maximum electric power of 1.18 W, featuring both sensing and energy supply functions. It is particularly suitable for remote infrastructure monitoring scenarios such as remote tunnels, where wiring is difficult.

As shown in Figure 8c, Guo et al. [112] proposed a cable vibration energy harvesting network based on CCT-TEHG. By adopting soft-contact structural designs such as a rabbit hair-coated CCT-TEHG, the environmental adaptability of the device is effectively improved, interface wear is significantly reduced, and the service life is prolonged. This system can construct a scalable distributed monitoring network to realize real-time anomaly detection and condition identification for key components such as cables, promoting the transformation of traditional infrastructure toward energy-self-sufficient intelligent civil structures. As shown in Figure 8d, Wang et al. [113] proposed a hybrid energy system integrating a TENG and solar cells (SC). Among them, the TENG harvests wind energy through wind-induced vibrations of Kapton/Cu/FEP films, while the solar cells capture solar energy. Through reasonable impedance matching design, the two form a wind–solar complementary energy harvesting mode, with power densities of 26 mW and 8 mW obtained from the TENG and SC, respectively. This composite structure effectively improves the stability and continuity of the energy output, showing good application potential in scenarios such as urban rooftops.

As depicted in Figure 8e, Zhu et al. [114] further combined a bladeless wind turbine with a TENG and introduced digital twin and 5G communication technologies to construct a self-powered road condition monitoring system. This design innovatively utilizes aerodynamic effects to efficiently convert random and hard-to-utilize natural gust disturbances into stable electric energy. While achieving energy self-sufficiency, it completes the real-time perception and remote transmission of road conditions, providing a brand-new technical solution for the monitoring and operation of intelligent transportation infrastructure. By installing flexible or embedded TENG sensors at key structural components, it is possible to detect minor vibrations and stress anomalies and then transmit the data wirelessly to a central monitoring station for real-time alerts. With deeper integration into Internet of Things (IoT) platforms, TENGs are set to play an increasingly important role in the whole-life-cycle health management of infrastructure, driving the advancement of intelligent city development.

### 5.4. Intelligent Monitoring of Human Health

Wearable devices have been widely used in health and sports monitoring, yet battery endurance and wearing comfort remain major constraints. TENGs can harness mechanical energy generated by everyday human activities—such as walking, joint bending, and respiration—to provide self-powered sensing, ensuring continuous operation and data collection for wearables, thereby significantly enhancing the user experience.

**Figure 9:**
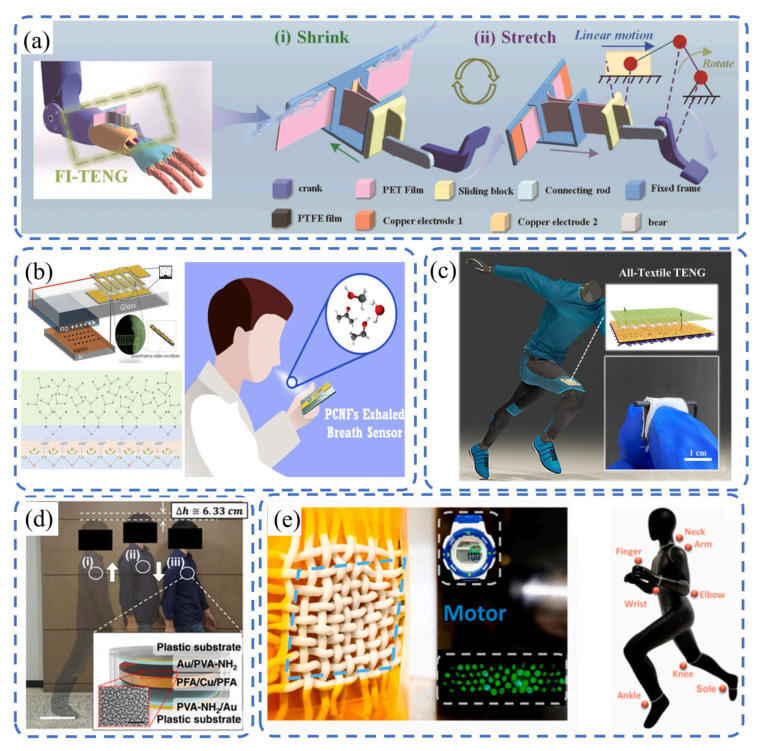
Applications of TENGs in human health monitoring. (**a**) Fountain-inspired TENG as rotary energy harvester and self-powered intelligent sensor [115], (**b**) self-powered PCNF-based exhaled breath sensor [116], (**c**) high-performance all-textile TENG toward intelligent sports sensing and biomechanical energy harvesting [117], (**d**) self-powered cardiac pacing system based on TENG [118], (**e**) flexible and stretchable TENG fabric for biomechanical energy harvesting and self-powered dual-mode human motion monitoring [119].

As illustrated in Figure 9a, Yin et al. [115] proposed a machine learning-assisted fountain-like TENG (FI-TENG). Its continuous sliding structure can amplify the displacement and positive pressure of the triboelectric layer; upon optimizing the triangular displacement amplification angle, gap width, and thickness of the PET film, the performance of the optimal scheme was 70% higher than that of the worst scheme. A crank-slider mechanism is adopted to adapt to human movement. In the wrist joint test (rotation angle 60°, frequency 1 Hz, load resistance 80 MΩ), the maximum output power density reached 64.65 mW/m^2^. Combined with the random forest (RF) and MobileNetV3-Small neural network, it achieved motion recognition with average accuracy of 97.56%, showing potential applications in telemedicine, rehabilitation assistance, and humanoid robots. As shown in Figure 9b, Mohamadbeigi et al. [116] integrated a contact separation triboelectric nanogenerator with a sensing unit to construct a self-powered PCNF-based exhaled breath sensor, enabling disease diagnosis and condition monitoring through changes in the concentrations of specific biomarkers. Under conditions of 90% relative humidity and the presence of interfering gases, the sensor demonstrated excellent response performance for 5 ppm and 200 ppm ethanol, with the response values reaching 0.9 and 3.2, respectively. It also exhibited outstanding ethanol selectivity, with selectivity ratios of 10:1 and 25:1 for methanol and acetone, respectively. In a 90% humidity environment, the sensor’s response and recovery times for 200 ppm ethanol were as fast as 2.7 s and 5.8 s, showcasing rapid response characteristics. Practical tests confirmed the sensor’s stable and reliable performance, highlighting its potential for integration into wearable devices for the continuous monitoring of lung cancer-related biomarkers, as well as for assisting in the compliance testing of legal alcohol intake limits.

As shown in Figure 9c, Zheng et al. [117] proposed a highly flexible and cuttable single-electrode all-textile TENG (t-TENG). Through dielectric modulation to optimize charge storage and offset charge dissipation, the micro-device (2 × 2 cm^2^, 130 mg) achieved outputs of 261 V, 1.5 μA, and 12.7 nC, with an instantaneous power density of 654.48 mW·m^−2^. It exhibited excellent stability in 20,000 cycle tests and high sensitivity of 3.438 V·kPa^−1^ in the force range of 1–10 N. It can light up more than 110 LEDs and can be integrated into clothing as a wearable power supply, showing great application potential in the field of intelligent motion sensing. As depicted in Figure 9d, Ryu et al. [118] reported a high-performance inertia-driven TENG (I-TENG) with the size of a commercial button battery. It has a closed five-layer stacked structure and can convert mechanical energy into electrical energy, with root mean square output power of 4.9 μW/cm^3^. In preclinical trials, it could monitor the output voltage via Bluetooth and charge lithium-ion batteries. After integration with a cardiac pacemaker, the ventricular pacing and sensing modes of this self-charging cardiac pacemaker system were verified, paving the way for the development of new self-charging implantable medical devices.

As shown in Figure 9e, He et al. [119] designed a flexible and stretchable coaxial TENG yarn, with a spiral spring as the inner support layer and a mechanoluminescent ZnS:Cu/PDMS composite material as the outer triboelectric layer. Weaving or blending can produce multifunctional TENG fabrics, which can be used to collect mechanical energy from human movement and the surrounding environment. Moreover, it can realize self-powered human motion sensing through two modes, electrical (via TENG) and optical (from mechanoluminescent materials), showing potential applications in long-term medical monitoring and human–computer interaction systems. To address the issue of traditional hydrogel TENGs being prone to freezing at low temperatures and losing water at high temperatures, leading to unstable performance, Wu et al. [120] designed an anti-freezing eutectic gel based on a deep eutectic solvent to enhance the stability and conductivity of TENGs in extreme environments. The fabricated eutectic gel-based TENG (E-TENG) achieved an open-circuit voltage of 776 V, a short-circuit current of 1.54 μA, and a peak power output of 1.1 mW, while exhibiting excellent mechanical properties with fracture elongation of 476%. The device can operate stably across a wide temperature range of −18 to 60 °C, with conductivities of 2.15 S/m and 1.75 S/m at −10 °C and −18 °C, respectively. Thanks to its outstanding weight stability, the E-TENG can not only monitor motion in air but also function underwater. When integrated with a Bluetooth module, this self-powered E-TENG can serve as a wearable sensor to detect human motion signals in real time and wirelessly transmit health data to mobile devices. This work opens up new avenues for the application of TENGs in extreme environments and provides a feasible solution for wireless flexible sensors capable of real-time health monitoring.

Combining TENGs with modules for heart rate, electromyography, body temperature, and other physiological signals can create lightweight, self-powered intelligent health monitoring systems capable of long-term continuous tracking. With advances in flexible materials and micro-/nanofabrication techniques, TENG applications in wearable devices will become more skin-conformable and adaptable to diverse motion scenarios, offering innovative pathways for personalized healthcare and remote health management.

### 5.5. IoT Wireless Intelligent Monitoring

Against the backdrop of the deep integration of IoT technologies across industries, wireless intelligent sensing—as the core enabler of the IoT perception layer—is increasingly demanded in large-scale applications across critical fields such as marine meteorology, agricultural environments, and industrial equipment maintenance. Triboelectric nanogenerators and hybrid power generation technologies have provided a novel technical pathway for the self-powered development of IoT wireless intelligent sensing nodes, enabling an autonomous energy supply and the long-term stable operation of these nodes. This breakthrough serves as fundamental support for the lightweight, low-power, and distributed deployment of IoT wireless intelligent sensing systems.

Cui et al. [121] developed an ultra-high-output hybrid triboelectric–electromagnetic generator with a swing mechanism, establishing a self-powered wireless sensor network integrating a power management system, meteorological sensors, and LoRa wireless transmission to construct an ocean meteorological monitoring system for the real-time in situ measurement of the temperature, humidity, and air pressure in marine areas. Xu et al. [122] designed a fluorinated ethylene propylene (FEP)-based dual-TENG-driven room-temperature humidity sensor. Using a composite of chitosan and activated carbon as the humidity-sensitive material, they prepared a humidity-sensitive film on a nichrome alloy interdigitated electrode substrate via a spraying process. The TENG exhibited a peak voltage of nearly 700 V and maximum output power of 12.208 mW. After regulation by rectification and operational amplifier circuits, it achieved a wide-range humidity response of 0–48 V, covering a detection range of 0–97% RH, with excellent response recovery characteristics and operational stability. It can accurately identify human respiratory patterns, finger proximity, and liquid surface coverage changes, transmitting sensor signals to smart terminals via a WiFi module and performing respiratory data collection and analysis. Gu et al. [123] developed a self-powered multi-channel agricultural wireless sensing system based on a pulsed triboelectric nanogenerator using a corn husk composite film, capable of simultaneously collecting various parameters, such as the farmland temperature, humidity, light intensity, and soil moisture, supporting wind energy applications and being suitable for intelligent green agriculture environmental monitoring.

Wu et al. [124] proposed an integrated vibration sensing system combining an instantaneous discharge-enhanced triboelectric nanogenerator with infrared wireless communication, utilizing a sandwich-structured TENG to achieve ultra-high instantaneous power output and integrating an infrared emission module to build a wireless monitoring system for online vibration state monitoring and the visual characterization of equipment. Zhang et al. [125] developed a TENG-driven tip-to-tip electrode air discharge switch (T-TADS). Compared to traditional mechanical and electronic switches, it offers advantages such as a lightweight design, easy integration, strong voltage adaptability, and stable discharge performance, increasing the instantaneous output power for external resistances below 1 MΩ by 1600 times. When paired with a passive power management circuit, its energy storage efficiency reaches 58.9%. Further incorporating a gas discharge tube (GDT) with a stable breakdown voltage, compact size, and weight of only about 0.9 g, they verified its electrical properties, which were similar to those of the air discharge switch, achieving optimal energy storage efficiency of 56.3%. Based on a wind-driven TENG and a power management circuit with a GDT, they developed a self-powered wireless meteorological sensing system that transmits temperature, humidity, atmospheric pressure, and light intensity data every 11 min, with a maximum range of 800 m, making it suitable for environmental monitoring in remote, off-grid areas such as high-altitude regions, deserts, and mountains.

To address key challenges such as self-powering, wireless transmission and precise sensing in diverse scenarios, researchers have conducted systematic and innovative studies. Breakthroughs have been achieved in the structural design of self-powered sensing nodes, the optimization of energy management efficiency, the integration of wireless transmission, and multi-scenario adaptability. These advancements effectively resolve critical technical issues related to energy supply stability, monitoring accuracy, transmission performance, and environmental adaptability in IoT wireless intelligent sensing systems. The research outcomes provide practical technical solutions for intelligent, distributed, and eco-friendly sensing in marine meteorological monitoring, smart agriculture, and industrial equipment maintenance under the IoT framework. They also lay a solid foundation for the large-scale deployment and future innovation of IoT wireless intelligent sensing systems.

## 6. Discussion

**Figure 10:**
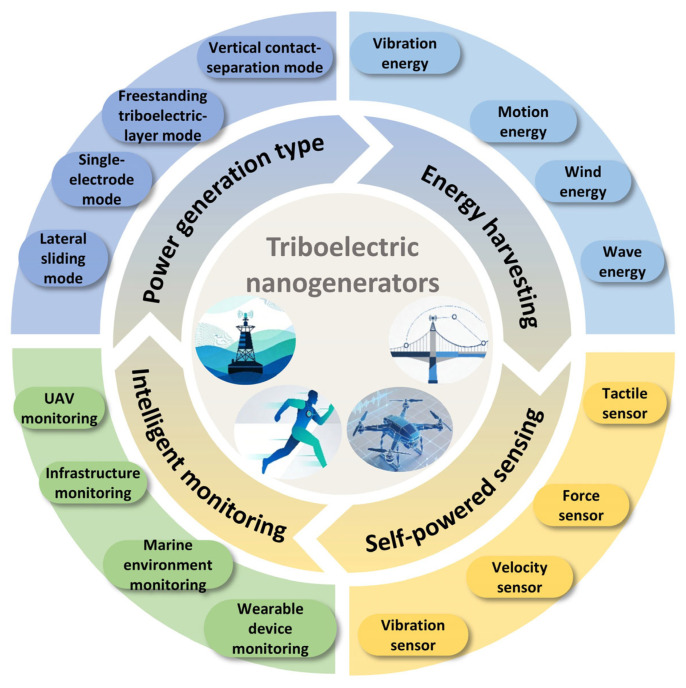
TENGs enable significant progress in energy harvesting, self-powered sensing, and intelligent monitoring.

Although TENGs have achieved significant progress in energy harvesting, self-powered sensing, and intelligent monitoring, as shown in Figure 10, several challenges remain in this research field. This section discusses the challenges faced and future research directions as follows.

1. Theoretically, while mechanisms for various operating modes—contact separation, lateral sliding, single electrodes, and independent layers—have been preliminarily established, the precise modeling of multi-physics coupling under complex conditions remains inadequate. Existing models exhibit limited analytical capabilities regarding charge transfer at material interfaces, dynamic air gap variations, and high-frequency nonlinear responses, resulting in performance enhancement strategies lacking robust theoretical foundations. On one hand, maintaining high-charge-density generation while reducing friction losses is crucial in enhancing the micro-energy harvesting capacity and extending device lifespans. This requires in-depth research in material surface modification, interface engineering, and triboelectric sequence matching. On the other hand, the inherent high output impedance of TENGs limits the energy extraction efficiency. Achieving low-impedance matching and efficient power management while ensuring a high-voltage output demands breakthroughs in circuit theory and impedance transformation techniques.

2. Regarding structural design, most existing vibration energy harvesters and sensors follow the traditional inertial mass paradigm, relying on spring–mass systems or cantilever beam structures to enhance mechanical vibration capture. While effective within specific frequency bands, such designs often suffer from structural complexity, bulky dimensions, and significant weight, failing to meet miniaturization and lightweight requirements. Particularly on platforms like drones and wearable devices, where payload capacity and spatial constraints are critical, rigid, heavy sensors not only hinder conformal integration but also consume valuable payload space, compromising the aerodynamic and dynamic performance. This severely limits flexible deployment and structural innovation within confined spaces. Therefore, there is an urgent need to develop novel lightweight, flexible, and conformable structural solutions, such as compact topologies based on origami, thin films, and rolling friction, to achieve truly integrated and space-friendly designs.

3. Regarding energy storage and output stability, existing TENG energy harvesters primarily target real-time applications. Their output voltage is significantly affected by random environmental energy fluctuations, leading to unstable outputs. Due to the uncontrollable amplitudes and frequencies of natural vibration sources, relying solely on the harvesting end makes it difficult to achieve a continuous and stable power supply. Energy storage has thus become essential for ensuring a stable output. Currently, the integration of TENGs with energy storage units like supercapacitors and thin-film batteries remains exploratory. Key technical challenges include achieving efficient energy buffering and smooth outputs under high internal resistance, as well as developing specialized energy management circuits tailored to TENGs’ characteristics.

4. In the field of real-time self-powered sensing and monitoring, a truly real-time self-powered system based on TENGs is still lacking. Due to the severe impedance mismatch between the high internal resistance of TENGs and the load, the highly intermittent energy supply caused by pulsed outputs, and the absence of efficient and low-power adaptive power management circuits, as well as sensitivity to environmental disturbances, material wear, and insufficient long-term stability, it is difficult to sustain the synchronous and stable operation of sensing, computation, and communication. Key challenges such as coordinated impedance regulation, a continuous and smooth energy output, system-level integration, and reliability engineering must be urgently addressed to achieve a truly battery-free, real-time, and self-sustaining energy system.

5. In the integration of self-powered monitoring and intelligent algorithms, current research largely remains confined to basic electrical signal acquisition and simple wireless transmission, lacking exploration of the data’s deeper value. Although TENG outputs contain rich temporal, frequency, and transient characteristics, cases deeply integrating wireless sensing with cutting-edge machine learning algorithms remain scarce. In advanced applications such as real-time attitude interpretation and precise structural damage localization, the absence of efficient feature extraction models and edge computing capabilities hinders the transformation of massive sensor data into intuitive and reliable status assessments. Furthermore, machine learning-based TENG inverse design—deriving materials, structures, and operating modes from target performance—holds significant promise for achieving device customization and modularization. This field remains in its infancy and requires interdisciplinary collaboration for advancement.

Overall, strategies for enhancing the integrated performance of TENGs in energy harvesting, sensing, and monitoring remain significantly underdeveloped. Future efforts should integrate materials, structures, hardware, and software within a unified design framework. Through multi-level, multidimensional optimization, the comprehensive performance of TENGs in energy harvesting, sensing, and monitoring can be systematically enhanced, overcoming the current engineering bottlenecks. This will ultimately enable deployable, highly reliable, and intelligent TENG systems capable of meeting the long-term application demands of complex scenarios such as intelligent cities, industrial IoT, and aerospace/maritime environments.

## 7. Conclusions

This paper provides a comprehensive review of the latest advancements in TENGs as a micro–nano energy harvesting and self-powered sensing technology across energy harvesting, self-powered sensing, and intelligent monitoring applications. In energy harvesting, research has achieved efficient energy capture in broadband, multidimensional, and random low-frequency vibration environments through structural topology optimization and innovative operational mechanisms. Representative structures include spring–mass systems, cantilever beams, rolling friction designs, and multi-layer stacked composites, adaptable to diverse vibration characteristics across scenarios. By integrating hybrid power generation modes and surface modification techniques, these approaches overcome the power density limitations of single-mechanism designs, validating the TENG’s feasibility as a distributed micro–nano energy source. In self-powered sensing, TENGs leverage the intrinsic functional relationships between electrical signals and mechanical excitation to achieve zero-static-power sensing. This covers the detection of multiple physical quantities, including vibration, rotational speed, displacement, and ocean wave parameters, while adapting to moving objects in single-electrode mode. Through deep integration with machine learning, sensing systems extract profound insights from time–frequency-domain features, enabling high-precision analysis in complex scenarios like sediment identification, attitude reconstruction, and bearing fault diagnosis. This marks a leap from data acquisition to knowledge generation in self-powered technologies. In the trend of intelligent monitoring, TENGs are synergizing with IoT, edge computing, and artificial intelligence to expand into scenarios like intelligent cities, Industry 4.0, and low-altitude economies. Their applications in drone status and fault monitoring, infrastructure intelligent monitoring, human health monitoring, and marine intelligent monitoring have established a “perception–assessment–prediction” closed-loop system.

## Data Availability

No new data were created or analyzed in this study.

## Supplementary Materials

The following supporting information can be downloaded at: https://www.mdpi.com/article/10.3390/s26102984/s1, Figure S1: Contact-separation triboelectric nanogenerator.

## References

[1] Liu Q., Wang Y., Zhao F., Zheng C., Xie J. (2025). A Review of the Research Progress of Sensor Monitoring Technology in Harsh Engineering Environments. Sensors.

[2] Wang Z.L. (2020). Triboelectric Nanogenerator (TENG)—Sparking an Energy and Sensor Revolution. Adv. Energy Mater..

[3] Thakur S., Sarkar N.I., Yongchareon S. (2025). AI-Driven Energy-Efficient Routing in IoT-Based Wireless Sensor Networks: A Comprehensive Review. Sensors.

[4] Silva R.R.A., Fatti G., Carlos E., Ferreira G., Goswami S., Nandy S., Mattoso L.H.C., Fortunato E., Otoni C.G., Martins R. (2026). Functional Materials for Environmental Energy Harvesting in Smart Agriculture via Triboelectric Nanogenerators. Adv. Funct. Mater..

[5] Wakshume D.G., Płaczek M.Ł. (2024). Optimizing Piezoelectric Energy Harvesting from Mechanical Vibration for Electrical Efficiency: A Comprehensive Review. Electronics.

[6] Gao Z., Zhou Y., Zhang J., Foroughi J., Peng S., Baughman R.H., Wang Z.L., Wang C.H. (2024). Advanced Energy Harvesters and Energy Storage for Powering Wearable and Implantable Medical Devices. Adv. Mater..

[7] Li H., Liang C., Ning H., Liu J., Zheng C., Li J., Yao H., Peng Y., Wan L., Liu G. (2022). O-ring-modularized triboelectric nanogenerator for robust blue energy harvesting in all-sea areas. Nano Energy.

[8] Du T., Dong F., Xi Z., Zhu M., Zou Y., Sun P., Xu M. (2023). Recent Advances in Mechanical Vibration Energy Harvesters Based on Triboelectric Nanogenerators. Small.

[9] Wu K., Fan C., Tang M., Chen H., Pan Y., Luo D., Zhang Z. (2025). Symbiotic energy-sensing wind generator enabled AI for smart roads. Sustain. Energy Fuels.

[10] Zhou H., Liu G., Zeng J., Dai Y., Zhou W., Xiao C., Dang T., Yu W., Chen Y., Zhang C. (2022). Recent Progress of Switching Power Management for Triboelectric Nanogenerators. Sensors.

[11] Naderpoor Shad P., Taghikhany T. (2026). Semi-active control of high-rise buildings with limited sensors: A dynamic neural network approach for simultaneous sensor and damper fault diagnosis. J. Sound Vib..

[12] Zhou Y. (2025). Unmanned aerial vehicles based low-altitude economy with lifecycle techno-economic-environmental analysis for sustainable and smart cities. J. Clean. Prod..

[13] Talaat F.M., Ibrahim M.A., Karim A.A., Elsonbaty H.K., Al-Zoghby A.M. (2026). IoT-Integrated robotic system for automated plant disease detection and environmental monitoring. Sci. Rep..

[14] Zhang B., Ren Y., He S., Gao Z., Li B., Song J. (2025). A review of methods and applications in structural health monitoring (SHM) for bridges. Measurement.

[15] D’Alessandro A., Birgin H.B., Cerni G., Ubertini F. (2022). Smart Infrastructure Monitoring through Self-Sensing Composite Sensors and Systems: A Study on Smart Concrete Sensors with Varying Carbon-Based Filler. Infrastructures.

[16] Wang C., Zhang X., Wu J., Yu X., Cheng T., Ma H., Wang Z.L. (2022). Double-spring-piece structured triboelectric sensor for broadband vibration monitoring and warning. Mech. Syst. Signal Process..

[17] Vavachan V.K., Joseph S., John H., Saji K.J. (2024). High-performance latex-compounded nitrile rubber for self-powered intelligent traffic monitoring with advanced signal conditioning. ACS Appl. Eng. Mater..

[18] Gu L., German L., Li T., Li J., Shao Y., Long Y., Wang J., Wang X. (2021). Energy Harvesting Floor from Commercial Cellulosic Materials for a Self-Powered Wireless Transmission Sensor System. ACS Appl. Mater. Interfaces.

[19] Jiang D., Lian M., Xu M., Sun Q., Xu B.B., Thabet H.K., El-Bahy S.M., Ibrahim M.M., Huang M., Guo Z. (2023). Advances in triboelectric nanogenerator technology—Applications in self-powered sensors, Internet of things, biomedicine, and blue energy. Adv. Compos. Hybrid Mater..

[20] Zhang W., Zhang X., Yu Y., Cheng X., Li H., Liu S., Meng L., Wang Z.L., Cheng T. (2023). Self-Powered Intelligent Damper Integrated Triboelectric-Electromagnetic Hybrid Unit for Vibration In Situ Monitoring of Stay Cables. Adv. Energy Mater..

[21] He Q., Briscoe J. (2024). Piezoelectric Energy Harvester Technologies: Synthesis, Mechanisms, and Multifunctional Applications. ACS Appl. Mater. Interfaces.

[22] Zhou Z., Xu Z., Cao L.N.Y., Sheng H., Li C., Shang Y., Tang W., Wang Z.L. (2024). Triboelectricity Based Self-Powered Digital Displacement Sensor for Aircraft Flight Actuation. Adv. Funct. Mater..

[23] Qi L., Song J., Wang Y., Yi M., Zhang Z., Yan J. (2024). Mechanical motion rectification-based electromagnetic vibration energy harvesting technology: A review. Energy.

[24] Zhou F., Wei L., Tang L., Shan M., Wang Z., Peng J., Zhang Z., Liu X., Zhang Q., Wang F. (2025). Nano-Interlocking Enhanced Electroactive Dressing: Electromagnetic Induction for Accelerated Diabetic Wound Healing and Wound Microenvironment Monitoring. Adv. Funct. Mater..

[25] Xu Z., Jiao Y., Dong P., Ji G., Chen S., Zheng J. (2026). High-performance, thermally stable and recyclable triboelectric nanogenerators benefit from triple-functional silyl ether networks interspersed with engineered MXene. Nano Energy.

[26] Zheng R., Zheng L., Li J., Yang Y., Zheng Q., Zhang L., Fan K., Yang R. (2026). A self-powered single-transistor synchronous switching strategy toward efficient utilization of triboelectric energy. Nano Energy.

[27] Liu W., Wang Z., Wang G., Liu G., Chen J., Pu X., Xi Y., Wang X., Guo H., Hu C. (2019). Integrated charge excitation triboelectric nanogenerator. Nat. Commun..

[28] Pan Y.C., Dai Z., Ma H., Zheng J., Leng J., Xie C., Yuan Y., Yang W., Yalikun Y., Song X. (2024). Self-powered and speed-adjustable sensor for abyssal ocean current measurements based on triboelectric nanogenerators. Nat. Commun..

[29] Zheng Y., Liu T., Wu J., Xu T., Wang X., Han X., Cui H., Xu X., Pan C., Li X. (2022). Energy Conversion Analysis of Multilayered Triboelectric Nanogenerators for Synergistic Rain and Solar Energy Harvesting. Adv. Mater..

[30] Xu J., Kong L., Wang Y., Hong H. (2025). Using deep learning and an annular triboelectric sensor for monitoring downhole motor rotor faults. Nano Energy.

[31] Dong Y., Duan L., Mao X., Gu T., Gu Y., Yu H., Zhang X., Ye T., Wang X., Li P. (2025). Exploring human motions for smart wearables: Energy conversion, harvesting and self–powered sensing. Nano Energy.

[32] Wang Y., Liu X., Chen T., Wang H., Zhu C., Yu H., Song L., Pan X., Mi J., Lee C. (2021). An underwater flag-like triboelectric nanogenerator for harvesting ocean current energy under extremely low velocity condition. Nano Energy.

[33] Liu Y., Wang J., Liu T., Wei Z., Luo B., Chi M., Zhang S., Cai C., Gao C., Zhao T. (2025). Triboelectric tactile sensor for pressure and temperature sensing in high-temperature applications. Nat. Commun..

[34] Yao C., Liu S., Liu Z., Huang S., Sun T., He M., Xiao G., Ouyang H., Tao Y., Qiao Y. (2025). Deep learning-enhanced anti-noise triboelectric acoustic sensor for human-machine collaboration in noisy environments. Nat. Commun..

[35] Zhang H., Zhang D., Yang Y., Zhou L., Liu Y., Liu W., Sun Y., Guo Y., Ji Y. (2024). Eco-friendly triboelectric nanogenerator for self-powering stacked In_2_O_3_ nanosheets/PPy nanoparticles-based NO_2_ gas sensor. Nano Energy.

[36] Wang L., Li S., Long J., Wang Y., Li H., Bian Z., Zheng L. (2025). Intelligent traffic speed bump based on triboelectric nanogenerator for vehicle overload detection, roadway behavior recognition, and traffic signal alarming. Nano Energy.

[37] Cui X., Du G., Liu T., Ye Z., Liu Y., Cai C., Luo B., Nie S. (2025). Cellulosic Triboelectric Elastomers for Energy Harvesting and Emerging Applications. Adv. Funct. Mater..

[38] Xia X., Fan C., Zhou Q., Kong W., Liu G., Zhang Z., Pan Y., Luo D., Azam A., Tang M. (2024). A self-powered and self-sensing wave energy harvester based on a three-rotor motor of axle disk type for sustainable sea. Energy.

[39] Yu J., Wen Y., Yang L., Zhao Z., Guo Y., Guo X. (2022). Monitoring on triboelectric nanogenerator and deep learning method. Nano Energy.

[40] Duan J., Lei Y., Liu Z., Song Y., Wu Y. (2026). TENG-Based Wearable Sensors for Continuous Self-Powered Health Monitoring. Adv. Sens. Res..

[41] Cui Y., Luo H., Yang T., Qin W., Jing X. (2025). Bio-inspired structures for energy harvesting self-powered sensing and smart monitoring. Mech. Syst. Signal Process..

[42] Luo H., Ni X., Cui Y., Huang C., Yuan P., Yang T., Shao J., Huang X. (2025). High stability rotary solid-liquid triboelectric nanogenerator for ionic liquid detection. Nano Energy.

[43] Luo H., Ni X., Zhang C., Cui Y., Yang T., Shao J., Jing X. (2024). Multi-Phase Rotating Disk Triboelectric Nanogenerator with DC Output for Speed Monitoring. Small.

[44] Luo H., Liu J., Yang T., Zhang Y., Cao Q. (2022). Dipteran flight-inspired bistable triboelectric nanogenerator for harvesting low frequency vibration. Nano Energy.

[45] Zhou S., Lallart M., Erturk A. (2022). Multistable vibration energy harvesters: Principle, progress, and perspectives. J. Sound Vib..

[46] Fang S., Zhang X., Fan J., Guan Y., Liu W., Cai M., Lai Z., Huang X., Wang X., Zhou S. (2026). A high-output dynamic bistable electromagnetic energy harvester for ultra-low-frequency rotational excitations. Mech. Syst. Signal Process..

[47] Liu J., Luo H., Yang T., Cui Y., Lu K., Qin W. (2024). Double bistable superposition strategy for improving the performance of triboelectric nanogenerator. Mech. Syst. Signal Process..

[48] Luo H., Luo B., Yang T., Ni X., Gao C., Song S., Luo S., Gao Y., Wang M., Zheng X. (2026). Corona discharge triboelectric nanogenerator with enhanced charge accumulation effect. Chem. Eng. J..

[49] Hou C., Tan J., Su P., Song H., Wu J., Xie T., Tan J., Xu J. (2026). Broadband performance of a rotating piezoelectric energy harvester with bending-torsion composite and magnetic nonlinearity. Mech. Syst. Signal Process..

[50] Liu D., Luo J., Huang L., Chen M., Ji M., Wang Z.L., Kang J. (2025). Triboelectric nanogenerators as a practical approach for wind energy harvesting: Mechanisms, designs, and applications. Nano Energy.

[51] Tang J., Shang Y., Meng J., Li J., Xu M., Hu Y., Zhang J. (2026). A new method for quantitative evaluation of micro-cracks on turbine blade surfaces Fusing triboelectric sensing and hybrid deep learning. Mech. Syst. Signal Process..

[52] Wang C., Yang Y., Zhang X., Wang P., Bi X., Li H., Wang Z.L., Cheng T. (2024). Ultra-High Sensitivity Real-Time Monitoring of Landslide Surface Deformation via Triboelectric Nanogenerator. Adv. Mater..

[53] Yang Y., Zhang S., Li K., Xue S., Cai T. (2025). All-in-one self-powered wind speed sensor with a wide start-up range and high output power. APL Mater..

[54] Zou Y., Xu J., Chen K., Chen J. (2021). Advances in Nanostructures for High-Performance Triboelectric Nanogenerators. Adv. Mater. Technol..

[55] Hou X., Zhou J., Shi R., Liu H., Zhang M., Guo Z., Bai Y., Li X., Gao X., Guo J. (2025). Triboelectric nanogenerator sensors-based trans-media motion monitoring system for Gannet-inspired vehicle aiming at digital twin applications. Nano Energy.

[56] Bairagi S., Zada M., Otesteanu C., Menon C. (2026). Electro-active phase assisted all-fiber triboelectric nanogenerator (AF-TENG) for energy harvesting and human joint angle monitoring. Nano Energy.

[57] Liu S., Guo J., Zhu Z., Meng L., Li X. (2026). Multi-degree-of-freedom energy-harvesting and monitoring-coupled triboelectric nanogenerator for vibration state perception of transmission towers. Nano Energy.

[58] Shahat A., Mahmoud M.A., El-Sewify I.M., Reda A., Akter N., Alharbi A., Radwan A., Hasan M., Shenashen M.A., El-Safty S.A. (2025). Nanogenerator-induced personalized wearable health monitoring electronics: A review. Nano Energy.

[59] Wang Z.L. (2022). On the expanded Maxwell’s equations for moving charged media system—General theory, mathematical solutions and applications in TENG. Mater. Today.

[60] Shao J., Jiang T., Wang Z. (2020). Theoretical foundations of triboelectric nanogenerators (TENGs). Sci. China Technol. Sci..

[61] Fan F.-R., Tian Z.-Q., Lin Wang Z. (2012). Flexible triboelectric generator. Nano Energy.

[62] Luo J., Gao W., Wang Z.L. (2021). The Triboelectric Nanogenerator as an Innovative Technology toward Intelligent Sports. Adv. Mater..

[63] Walden R., Kumar C., Mulvihill D.M., Pillai S.C. (2022). Opportunities and Challenges in Triboelectric Nanogenerator (TENG) based Sustainable Energy Generation Technologies: A Mini-Review. Chem. Eng. J. Adv..

[64] Cui Y., Yang T., Luo H., Li Z., Jing X. (2024). Jellyfish-inspired bistable piezoelectric-triboelectric hybrid generator for low-frequency vibration energy harvesting. Int. J. Mech. Sci..

[65] Yang X., Lai S.-K., Wang C., Wang J.-M., Ding H. (2022). On a spring-assisted multi-stable hybrid-integrated vibration energy harvester for ultra-low-frequency excitations. Energy.

[66] Qu M., Wei X., Liu H., Deng Y., Zhang R., Liu Z., Zhu M., Gao Y., Cao M., He J. (2025). Hybrid-Mode Triboelectric Nanogenerator Based on Cantilever Beam for Enhanced Droplet Energy Harvesting. ACS Appl. Electron. Mater..

[67] Lian Z., Wang Q., Zhu C., Zhao C., Zhao Q., Wang Y., Hu Z., Xu R., Lin Y., Chen T. (2022). A Cantilever Beam-Based Triboelectric Nanogenerator as a Drill Pipe Transverse Vibration Energy Harvester Powering Intelligent Exploitation System. Sensors.

[68] Wang K., Wu C., Zhang H., Li J., Li J. (2022). Cylindrical bearing inspired oil enhanced rolling friction based nanogenerator. Nano Energy.

[69] Pongampai S., Pakawanit P., Charoonsuk T., Vittayakorn N. (2021). Low-cost fabrication of the highly efficient triboelectric nanogenerator by designing a 3D multi-layer origami structure combined with self-charged pumping module. Nano Energy.

[70] Wu C., Wang A.C., Ding W., Guo H., Wang Z.L. (2019). Triboelectric Nanogenerator: A Foundation of the Energy for the New Era. Adv. Energy Mater..

[71] Zhang H., Dai G., Liu H., Xiongsong T., Liu Y., Liu Q., Gao M., Yin K., Yang J. (2026). Four-electrode tubular liquid-solid triboelectric nanogenerator constructed via synergistic effects with the electric double layer and the switching: Enabling AC/DC convertible outputs. Energy.

[72] Yin P., Tang L., Aw K.C., Xia C., Salman M., Zhang D., Peng Y., Li Z. (2026). Self-charge pumping circuit with high reverse-voltage diodes for stepwise charge enhancement in stack triboelectric nanogenerators. Energy Convers. Manag..

[73] Zhang Z., Gu G., Zhang W., Du Z., Cheng G. (2024). Triboelectric nanogenerator with enhanced charge density and limited open-circuit voltage for efficient power management and industrial environmental monitoring. Nano Energy.

[74] Jiang X., Chen M., Liu W., Li K., Xu S., Yu H. (2025). A configurable high-precision multi-parameter signal measurement method and circuit framework for triboelectric nanogenerator characterization. Nano Energy.

[75] Zhang J., Zhao Z., Wang J. (2026). Impedance matching rules and voltage-current/charge regulation strategies for direct-current triboelectric nanogenerator. Chem. Eng. J..

[76] Choi D., Lee Y., Lin Z.-H., Cho S., Kim M., Ao C.K., Soh S., Sohn C., Jeong C.K., Lee J. (2023). Recent Advances in Triboelectric Nanogenerators: From Technological Progress to Commercial Applications. ACS Nano.

[77] Wang Z.L. (2013). Triboelectric Nanogenerators as New Energy Technology for Self-Powered Systems and as Active Mechanical and Chemical Sensors. ACS Nano.

[78] Zhao X., Liu L., Yin D., Su Y., Hu T., Zheng Y., Yang T., Shi Q., Tao K. (2025). A Vehicle-to-Everything (V2X) Interaction System for Intelligent Transportation Based on Energy Harvesting–Sensing Cooperation Triboelectric Nanogenerator. ACS Appl. Electron. Mater..

[79] Pan Y., Wang G., Wang K. (2025). Frictional nanogenerators (TENGs) in medical health monitoring: A progress review. AIP Adv..

[80] Yang T., Xie J., Huang Z., Liu J., Luo H., Jing X. (2025). Bio-inspired vibration isolator with triboelectric nanogenerator for self-powered monitoring. Mech. Syst. Signal Process..

[81] Yao L., Zhang H., Jiang J., Zhang Z., Zheng X. (2022). Recent Progress in Sensing Technology Based on Triboelectric Nanogenerators in Dynamic Behaviors. Sensors.

[82] Abbas Z., Prasanna A.P.S., Anithkumar M., Bincy T.S., Hussain N., Kim S.J., Mobin S.M. (2024). Development of new amine-functionalized metal-organic framework for enhanced triboelectrification using first-principle theory of nanogenerator. Nano Energy.

[83] Li Y., Yu J., Wei Y., Wang Y., Feng Z., Cheng L., Huo Z., Lei Y., Sun Q. (2023). Recent Progress in Self-Powered Wireless Sensors and Systems Based on TENG. Sensors.

[84] Yang H., Liu W., Xi Y., Lai M., Guo H., Liu G., Wang M., Li T., Ji X., Li X. (2018). Rolling friction contact-separation mode hybrid triboelectric nanogenerator for mechanical energy harvesting and self-powered multifunctional sensors. Nano Energy.

[85] Zhang C., Liu L., Zhou L., Yin X., Wei X., Hu Y., Liu Y., Chen S., Wang J., Wang Z.L. (2020). Self-Powered Sensor for Quantifying Ocean Surface Water Waves Based on Triboelectric Nanogenerator. ACS Nano.

[86] Hao S., Jiao J., Chen Y., Wang Z.L., Cao X. (2020). Natural wood-based triboelectric nanogenerator as self-powered sensing for smart homes and floors. Nano Energy.

[87] An S., Pu X., Zhou S., Wu Y., Li G., Xing P., Zhang Y., Hu C. (2022). Deep Learning Enabled Neck Motion Detection Using a Triboelectric Nanogenerator. ACS Nano.

[88] Huang T., Sun W., Liao L., Zhang K., Lu M., Jiang L., Chen S., Qin A. (2023). Detection of Microplastics Based on a Liquid–Solid Triboelectric Nanogenerator and a Deep Learning Method. ACS Appl. Mater. Interfaces.

[89] Liu L., Zhao X., Hu T., Liang F., Guo B., Tao K. (2024). Deep-learning-assisted self-powered wireless environmental monitoring system based on triboelectric nanogenerators with multiple sensing capabilities. Nano Energy.

[90] Dong F., Yang H., Du H., Zhu M., Xi Z., Wang Y., Du T., Xu M. (2024). Triboelectric nanogenerator-embedded intelligent bearing with rolling ball defect diagnosis via signal decomposition and automated machine learning. Nano Energy.

[91] Xu J., Yin J., Fang Y., Xiao X., Zou Y., Wang S., Chen J. (2023). Deep learning assisted ternary electrification layered triboelectric membrane sensor for self-powered home security. Nano Energy.

[92] Su Y., Yin D., Zhao X., Hu T., Liu L. (2025). Exploration of Advanced Applications of Triboelectric Nanogenerator-Based Self-Powered Sensors in the Era of Artificial Intelligence. Sensors.

[93] Mohsan S.A., Khan M.A., Noor F., Ullah I., Alsharif M.H. (2022). Towards the Unmanned Aerial Vehicles (UAVs): A Comprehensive Review Drones [Online]. Drones.

[94] Wang K., Yao Y., Liu Y., Guan X., Yu Y., Wang J., Wang Y., Li T., Cheng T. (2024). Self-powered system for real-time wireless monitoring and early warning of UAV motor vibration based on triboelectric nanogenerator. Nano Energy.

[95] Pan Z., Wang K., Liu Y., Guan X., Chen C., Liu J., Wang Z., Li F., Ma G., Yao Y. (2025). Deep learning-enhanced safety system for real-time in-situ blade damage monitoring in UAV using triboelectric sensor. Nano Energy.

[96] Guan X., Yao Y., Wang K., Liu Y., Pan Z., Wang Z., Yu Y., Li T. (2024). Wireless Online Rotation Monitoring System for UAV Motors Based on a Soft-Contact Triboelectric Nanogenerator. ACS Appl. Mater. Interfaces.

[97] Wang Z., Wang K., Liu Y., Guan X., Pan Z., Yao Y., Li T. (2025). Triboelectric Sensor with a Hierarchical Structure for Omnidirectional Adaptive Wind Speed and Wind Direction Sensing for Unmanned Aerial Vehicles. ACS Appl. Mater. Interfaces.

[98] Lu X., Zhong S., Zhou C., Tian S., Zhou W., Zheng Q., Li L., Jin T., Zhang Q., Zhang R. (2025). Self-powered real-time fault monitoring for drone blades. Nano Energy.

[99] Xie X., Chen Y., Jiang J., Li J., Yang Y., Liu Y., Yang L., Tu X., Sun X., Zhao C. (2021). Self-Powered Gyroscope Angle Sensor Based on Resistive Matching Effect of Triboelectric Nanogenerator. Adv. Mater. Technol..

[100] Khoshnoud F., Esat I.I., de Silva C.W., Rhodes J.D., Kiessling A.A., Quadrelli M.B. (2019). Self-Powered Solar Aerial Vehicles: Towards Infinite Endurance UAVs. Unmanned Syst..

[101] Wu X., Gao N., Zheng X., Tao X., He Y., Liu Z., Wang Y. (2020). Self-Powered and Green Ionic-Type Thermoelectric Paper Chips for Early Fire Alarming. ACS Appl. Mater. Interfaces.

[102] Chao X., Hu H., Lin J., Ge X., Wang Y., Xie L., Hu B., Meng L., Yu S., Liang F. (2025). Bioinspired twist-hyperbolic metamaterial for impact buffering and self-powered real-time sensing in UAVs. Sci. Adv..

[103] Hosseini S., Mesbahi M. (2016). Energy-Aware Aerial Surveillance for a Long-Endurance Solar-Powered Unmanned Aerial Vehicles. J. Guid. Control Dyn..

[104] Gupta A., Gupta S.K. (2022). A survey on green unmanned aerial vehicles-based fog computing: Challenges and future perspective. Trans. Emerg. Telecommun. Technol..

[105] Zhang C., Liu J., Shao Y., Ni X., Xie J., Luo H., Yang T. (2025). Rotational Triboelectric Nanogenerator with Machine Learning for Monitoring Speed. Sensors.

[106] Liu W., Li Y., Tang H., Zhang Z., Wu X., Zhao J., Zeng L., Tang M., Hao D. (2024). The nexus of sustainable fisheries: A hybrid self-powered and self-sensing wave energy harvester. Ocean Eng..

[107] Jiang Y., Chen P., Han J., Liang X., Ming Y., Liu S., Jiang T., Wang Z.L. (2025). High-performance triboelectric nanogenerator based on a double-spiral zigzag-origami structure for continuous sensing and signal transmission in marine environment. Interdiscip. Mater..

[108] Guan Z., Liu L., Xu X., Liu A., Wu H., Li J., Ou-Yang W. (2022). A self-powered acoustic sensor excited by ultrasonic wave for detecting and locating underwater ultrasonic sources. Nano Energy.

[109] Mao X., Zhang J., Duan L., Lyu B., Dong Y., Cao F., Jia C., Liu L., Chang H., Li Z. (2025). AI-driven ocean monitoring with multimodal triboelectric nanogenerator: Self-sustainable real-time wave warning and forecasting system. Nano Energy.

[110] Feng J., Zhou H., Cao Z., Zhang E., Xu S., Li W., Yao H., Wan L., Liu G. (2022). 0.5 m Triboelectric Nanogenerator for Efficient Blue Energy Harvesting of All-Sea Areas. Adv. Sci..

[111] Xu J., Wei X., Li R., Kong S., Wu Z., Wang Z.L. (2022). A Capsule-Shaped Triboelectric Nanogenerator for Self-Powered Health Monitoring of Traffic Facilities. ACS Mater. Lett..

[112] Guo X., Liu S., Zhang T., Zhu Z., Meng L. (2024). Cable Cross-Tie Triboelectric-Electromagnetic Hybrid Generator Triggered by Vibrations in Bridge Cables. Adv. Sustain. Syst..

[113] Du T., Dong F., Xu R., Zou Y., Wang H., Jiang X., Xi Z., Yuan H., Zhang Y., Sun P. (2022). A Drill Pipe-Embedded Vibration Energy Harvester and Self-Powered Sensor Based on Annular Type Triboelectric Nanogenerator for Measurement while Drilling System. Adv. Mater. Technol..

[114] Zhu M., Zhu J., Zhu J., Zhao Z., Li H., Cheng X., Wang Z.L., Cheng T. (2024). Bladeless Wind Turbine Triboelectric Nanogenerator for Effectively Harvesting Random Gust Energy. Adv. Energy Mater..

[115] Yin G., Liang X., Zhang Y., Li J., Wei S. (2025). Fountain-inspired triboelectric nanogenerator as rotary energy harvester and self-powered intelligent sensor. Nano Energy.

[116] Mohamadbeigi N., Shooshtari L., Fardindoost S., Vafaiee M., Iraji zad A., Mohammadpour R. (2024). Self-powered triboelectric nanogenerator sensor for detecting humidity level and monitoring ethanol variation in a simulated exhalation environment. Sci. Rep..

[117] Zheng Z., Ma X., Lu M., Yin H., Jiang L., Guo Y. (2024). High-Performance All-Textile Triboelectric Nanogenerator toward Intelligent Sports Sensing and Biomechanical Energy Harvesting. ACS Appl. Mater. Interfaces.

[118] Ryu H., Park H.M., Kim M.K., Kim B., Myoung H.S., Kim T.Y., Yoon H.J., Kwak S.S., Kim J., Hwang T.H. (2021). Self-rechargeable cardiac pacemaker system with triboelectric nanogenerators. Nat. Commun..

[119] Sommermann P., Cartmell M.P. (2021). The dynamics of an omnidirectional pendulum harvester. Nonlinear Dyn..

[120] Wu J., Teng X., Liu L., Cui H., Li X. (2024). Eutectogel-based self-powered wearable sensor for health monitoring in harsh environments. Nano Res..

[121] Cui W., Hu J., Yang H., Liu X., Wang Y., Lou Y., Li M., Li Z., Yu A., Wang Z.L. (2024). Self-powered wireless sensor node enabled by ultra-high-output swinging hybrid generator toward real-time and in-situ marine meteorological observations. Nano Energy.

[122] Xu Z., Zhang D., Liu X., Yang Y., Wang X., Xue Q. (2022). Self-powered multifunctional monitoring and analysis system based on dual-triboelectric nanogenerator and chitosan/activated carbon film humidity sensor. Nano Energy.

[123] Gu G., Gu G., Shang W., Zhang Z., Zhang W., Wang C., Fang D., Cheng G., Du Z. (2022). The self-powered agricultural sensing system with 1.7 km wireless multichannel signal transmission using a pulsed triboelectric nanogenerator of corn husk composite film. Nano Energy.

[124] Wu H., Wang Z., Zhu B., Wang H., Lu C., Kang M., Kang S., Ding W., Yang L., Liao R. (2023). All-in-One Sensing System for Online Vibration Monitoring via IR Wireless Communication as Driven by High-Power TENG. Adv. Energy Mater..

[125] Zhang W., Gu G., Zhang Z., Ren H., Zhou H., Gui Y., Du Z., Cheng G. (2024). Enhancing the output energy of triboelectric nanogenerator by adaptive arc discharge devices and its application in wireless weather sensing system. Nano Energy.

