# Supplementary material for: Triboelectric Nanogenerators Promote Self-Powered Sensing and Intelligent Monitoring
Source: Sensors (Basel). 2026 May 9;26(10):2984. doi: 10.3390/s26102984 (PMC13211271; doi:10.3390/s26102984)
Supplement: Supplementary file 1 [file sensors-26-02984-s001.zip › sensors-4249365-supplementary.pdf]

Supplementary Information

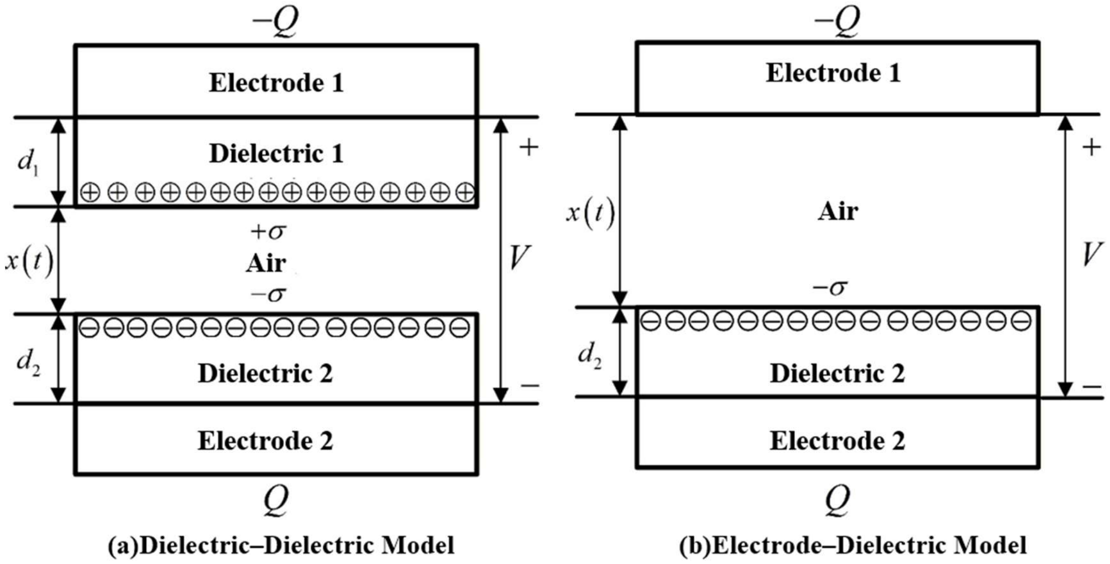

Supplementary Figure S1. Contact separation triboelectric nanogenerator. (a) Dielectric–dielectric model, (b) electrode–dielectric model.

Taking the contact separation triboelectric nanogenerator as an example, as shown in Supplementary Figure S1 (a), two types of dielectrics are used, with dielectric constants of ε_1 and ε_2; the thicknesses are, respectively, d_1 and d_2. When the two dielectrics come into physical contact, due to the contact electrification effect, static charges are transferred to their surfaces, partially charging them. These charges are immobile static charges. Surface charge density $\sigma_c(t)$ gradually accumulates with the contact between the two, eventually reaching saturation. The electrostatic field generated by frictional charges drives electrons to flow through an external load, resulting in the accumulation of free electrons in the electrodes σ. At this moment, the amount of charge transferred, driven by the induced electromotive force, is Q. At the same time, it also represents the instantaneous charge on the two electrodes. The electric fields in the two dielectrics between the electrodes and the field in the gap are, respectively,

$$E_1 = \frac{Q}{S\varepsilon_0\varepsilon_1}, \tag{S1}$$

$$E_2 = -\frac{Q}{S\varepsilon_0\varepsilon_2}, \tag{S2}$$

$$E_z = \frac{-\frac{Q}{S} + \sigma(t)}{\varepsilon_0}. \tag{S3}$$

The relative voltage between the two electrodes is expressed as

$$V = E_1 d_1 + E_1 d_1 + E_z x = -\frac{Q}{S\varepsilon_0}\left(\frac{d_1}{\varepsilon_1} + \frac{d_2}{\varepsilon_2} + x(t)\right) + \frac{\sigma x(t)}{\varepsilon_0}. \tag{S4}$$

For the metal conductor–dielectric model, the upper metal conductor serves both as a triboelectric layer and as an electrode. Therefore, the total charge in the metal conductor consists of two parts: one part is the triboelectric charge $S\sigma$, and the other part is the charge transferred between the two electrodes $-Q$. Hence, the total charge of the metal conductor is $S\sigma - Q$. Consequently, the relative voltage of the metal conductor–dielectric model can be expressed as

$$V = E_2 d_2 + E_z x = -\frac{Q}{S\varepsilon_0}\left(\frac{d_2}{\varepsilon_2} + x(t)\right) + \frac{\sigma x(t)}{\varepsilon_0}. \tag{S5}$$

Convert Eq. (S4) and Eq. (S5) into a unified form

$$V = -\frac{Q}{S\varepsilon_0}\left(d_0 + x(t)\right) + \frac{\sigma x(t)}{\varepsilon_0}, \tag{S6}$$

where d_0 is referred to as the effective thickness. For the dielectric–dielectric model，$d_0 = \frac{d_1}{\varepsilon_1} + \frac{d_2}{\varepsilon_2}$. For the metal conductor–dielectric model, $d_0 = \frac{d_2}{\varepsilon_2}$.

Now, let us analyze the system's output characteristics under different load conditions. When the system is open-circuit, the transferred charge between the electrodes is zero. Therefore, the open-circuit voltage of the system V_{oc} is

$$V_{oc} = \frac{\sigma x(t)}{\varepsilon_0}. \tag{S7}$$

When the system is short-circuited, the open-circuit voltage $V = 0$, and the amount of charge transferred Q_{sc} and the short-circuit current I_{sc} are

$$Q_{sc} = \frac{S\sigma x(t)}{d_0 + x(t)}, \tag{S8}$$

$$I_{sc} = \frac{dQ_{sc}}{dt} = \frac{S\sigma d_0}{\left(d_0 + x(t)\right)^2}\frac{dx}{dt} = \frac{S\sigma d_0 u(t)}{\left(d_0 + x(t)\right)^2}. \tag{S9}$$

When the system is connected to a load with a resistance value of R, according to Ohm's law, Eq. (S6) can be rewritten as

$$V = IR = R\frac{dQ}{dt} = -\frac{Q}{S\varepsilon_0}\left(d_0 + x(t)\right) + \frac{\sigma x(t)}{\varepsilon_0}. \tag{S10}$$

When $t = 0$, the two electrodes are not separated. Therefore, the amount of charge transferred Q is 0. At this point, the boundary conditions of the system can be obtained as

$$Q(t = 0) = 0. \tag{S11}$$

Substituting Eq. (S11) into Eq. (S10), the charge quantity $Q(t)$ can be solved:

$$Q(t)=\sigma S-\sigma S e^{-\frac{1}{RS\varepsilon_0}\left(d_0 t+\int_0^t x(t)dt\right)}-\frac{\sigma d_0}{R\varepsilon_0}e^{-\frac{1}{RS\varepsilon_0}\left(d_0 t+\int_0^t x(t)dt\right)}\bullet\int_0^t e^{\frac{1}{RS\varepsilon_0}\left(d_0 z+\int_0^z x(z)dz\right)}dz\ .\tag{S12}$$

The output current $I(t)$ and output voltage $U(t)$ can be expressed as

$$\begin{aligned}I(t)=\frac{dQ}{dt}=&-\frac{\sigma d_0}{R\varepsilon_0}+\frac{\sigma\left(d_0+x(t)\right)}{R\varepsilon_0}e^{-\frac{1}{RS\varepsilon_0}\left(d_0 t+\int_0^t x(t)dt\right)}\\&+\frac{\sigma d_0}{R\varepsilon_0}\frac{d_0+x(t)}{RS\varepsilon_0}e^{-\frac{1}{RS\varepsilon_0}\left(d_0 t+\int_0^t x(t)dt\right)}\\&\times\int_0^t e^{\frac{1}{RS\varepsilon_0}\left(d_0 z+\int_0^z x(z)dz\right)}dz.\end{aligned}\tag{S13}$$

$$\begin{aligned}U(t)=RI(t)=&-\frac{\sigma d_0}{\varepsilon_0}+\frac{\sigma\left(d_0+x(t)\right)}{\varepsilon_0}e^{-\frac{1}{RS\varepsilon_0}\left(d_0 t+\int_0^t x(t)dt\right)}\\&+\frac{\sigma d_0}{\varepsilon_0}\frac{d_0+x(t)}{S\varepsilon_0}e^{-\frac{1}{RS\varepsilon_0}\left(d_0 t+\int_0^t x(t)dt\right)}\\&\times\int_0^t e^{\frac{1}{RS\varepsilon_0}\left(d_0 z+\int_0^z x(z)dz\right)}dz.\end{aligned}\tag{S14}$$
